# Statin prescribing for people with severe mental illnesses: a staggered cohort study of ‘real-world’ impacts

**DOI:** 10.1136/bmjopen-2016-013154

**Published:** 2017-03-07

**Authors:** R Blackburn, D Osborn, K Walters, M Falcaro, I Nazareth, I Petersen

**Affiliations:** 1 Institute for Health Informatics, UCL, London, UK; 2 Division of Psychiatry, UCL, London, UK; 3 Primary Care and Population Health, UCL, London, UK

**Keywords:** CARDIOLOGY, MENTAL HEALTH, EPIDEMIOLOGY

## Abstract

**Objectives:**

To estimate the ‘real-world effectiveness of statins for primary prevention of cardiovascular disease (CVD) and for lipid modification in people with severe mental illnesses (SMI), including schizophrenia and bipolar disorder.

**Design:**

Series of staggered cohorts. We estimated the effect of statin prescribing on CVD outcomes using a multivariable Poisson regression model or linear regression for cholesterol outcomes.

**Setting:**

587 general practice (GP) surgeries across the UK reporting data to The Health Improvement Network.

**Participants:**

All permanently registered GP patients aged 40–84 years between 2002 and 2012 who had a diagnosis of SMI. Exclusion criteria were pre-existing CVD, statin-contraindicating conditions or a statin prescription within the 24 months prior to the study start.

**Exposure:**

One or more statin prescriptions during a 24-month ‘baseline’ period (vs no statin prescription during the same period).

**Main outcome measures:**

The primary outcome was combined first myocardial infarction and stroke. All-cause mortality and total cholesterol concentration were secondary outcomes.

**Results:**

We identified 2944 statin users and 42 886 statin non-users across the staggered cohorts. Statin prescribing was not associated with significant reduction in CVD events (incident rate ratio 0.89; 95% CI 0.68 to 1.15) or all-cause mortality (0.89; 95% CI 0.78 to 1.02). Statin prescribing was, however, associated with statistically significant reductions in total cholesterol of 1.2 mmol/L (95% CI 1.1 to 1.3) for up to 2 years after adjusting for differences in baseline characteristics. On average, total cholesterol decreased from 6.3 to 4.6 in statin users and 5.4 to 5.3 mmol/L in non-users.

**Conclusions:**

We found that statin prescribing to people with SMI in UK primary care was effective for lipid modification but not CVD events. The latter finding may reflect insufficient power to detect a smaller effect size than that observed in randomised controlled trials of statins in people without SMI.

**Strengths and limitations of this study**

- We used a large and representative data source that records real-world clinical data on cardiovascular outcomes and lipid modification in people with severe mental illness.
- Such data are both costly and difficult to obtain from other data sources and recruitment of people with severe mental illness into clinical trials may be challenging and result in substantial selection bias.
- We applied specialist methods to reduce the impact of confounding by indication (staggered cohort and multivariable regression) and handle missing covariate data (multiple imputation) within our study.
- However, these methods can only reduce bias arising from differences in measured confounders. Residual confounding may remain due to unmeasured confounders such as diet or severity of mental illness.
- We were not able to examine the association between statin prescribing and lipid modification among people who had missing data on total cholesterol at baseline.

## Introduction

People with severe mental illness (SMI) including schizophrenia and bipolar disorder are at a twofold to threefold higher risk of cardiovascular disease (CVD) than comparable individuals without SMI.^1^ ^2^ CVD drives a substantial portion of the 13–30 years deficit in life expectancy relative to the general population^3^ and National Institute for Health and Care Excellence (NICE) guidelines identify people with SMI as a population for which statin prescribing should be considered.^4–6^ Statins are cost-effective for preventing CVD events within randomised controlled trial (RCT) populations without mental illness.^7^ However, we do not know whether statins are similarly effective in people with SMI, since this population has differences in CVD risk profile (such as very high rates of smoking, obesity and diabetes). Furthermore, people with SMI may adhere to medication differently and have additional exposure to antipsychotic medications, which are associated with increased risk of dyslipidaemia.

We do not know if results from existing RCTs can be extrapolated to real-world impacts of statin prescribing to people with SMI. Over two-thirds of the participants in the most recent meta-analysis of statins for primary prevention of CVD^7^ were derived from studies that explicitly excluded participants with psychological conditions (50%; n=28 390)^8–10^ or individuals who were perceived as less likely to be compliant with treatment (20%; n=10 797).^11–13^

Few studies have assessed statin medication adherence in people with SMI and we are unaware of any that focus exclusively on primary CVD prevention. However, a small number of important studies have investigated cardiovascular medication adherence for combined primary and secondary prevention and report comparable or better adherence for people with schizophrenia relative to controls.^14^ ^15^ Despite such findings, mental illness is sometimes perceived as a barrier to good medication adherence^16–18^ and might therefore deter general practitioners from prescribing statins to people with SMI.^19^ Indeed, significant inequalities in statin prescribing have been reported for people with SMI.^20^ In addition, in vitro studies indicate that some antipsychotic agents interact with transcription factors for enzymes involved in cholesterol and fatty acid synthesis (including 3-hydroxy-3-methylglutaryl-coenzyme A (HMG-CoA) reductase, the primary binding site for statins) and could therefore counteract the cholesterol-lowering action of statins.^21^ ^22^ The degree of interaction appears to vary by type of antipsychotic and is more strongly correlated with agents such as clozapine, which are associated with weight gain.^23^ ^24^

We aimed to estimate the real-world impact of statin prescribing on people with SMI by comparing people with SMI who did (statin users), or did not (statin non-users), receive a statin prescription. We estimated the effect of statin prescribing on:

1. Combined first myocardial infarction (MI) and stroke (primary outcome);
2. All-cause mortality;
3. Change in total cholesterol concentration at 1 and 2 years after initiating a statin.

## Methods

### Study design

We used data from The Health Improvement Network (THIN), which captures anonymised data from electronic health records from over 12 million patients registered at 587 general practice (GP) surgeries (∼5.7% of the UK population).^25^ See end of article for details of ethical approval. This data source has been used extensively to investigate CVD risk in people with SMI.^1^ ^26^ ^27^ Individuals with SMI routinely access primary care^28^ and the validity of SMI diagnoses in computer records has been established.^29^ Furthermore, the incidence of SMI has been found to be comparable to other epidemiological studies.^30^ THIN data are recorded as hierarchical medical codes (Read codes), free-text comments, drug codes for prescribed medications, referrals and additional health information such as laboratory test results.^31^ ^32^ The Townsend score for the quintile of deprivation for a patient's address reflects deprivation (employment, household occupancy, car and home ownership as recorded in the 2001 census) at enumeration district level, which covers areas of ∼150 households.^33^

Classification criteria (including case definitions for SMI, CVD and statin prescribing) for this study are outlined in online supplementary file S1 and code lists are available on request from the authors. We included all people with a diagnosis of schizophrenia or bipolar disorder who were aged 40–84 years and consulted their GP between 1 January 2002 and 31 December 2013. We excluded patients who were not permanently registered, and data for time periods before the practice acceptable mortality rate, acceptable computer usage dates^34^ ^35^ and for the 12 months after patient registration.^36^ We excluded individuals who—before the cohort start—had any of: CVD diagnosis, statin prescription in the prior 24 months, condition likely to impact on initiating a statin (terminal illness, dementia or raised alanine transaminase and aspartate transaminase; defined as >3x upper limit of normal range; 124 and 116 IU/L, respectively).

10.1136/bmjopen-2016-013154.supp1 supplementary data

As described more fully in our study protocol (see online supplementary file S2), we used a staggered cohort study design to reduce the impact of confounding by indication.^37^ This type of study design has successfully been applied to other studies comparing an active (including statins for primary CVD) and passive treatment groups.^38–40^

10.1136/bmjopen-2016-013154.supp2 supplementary data

**Figure 1:**
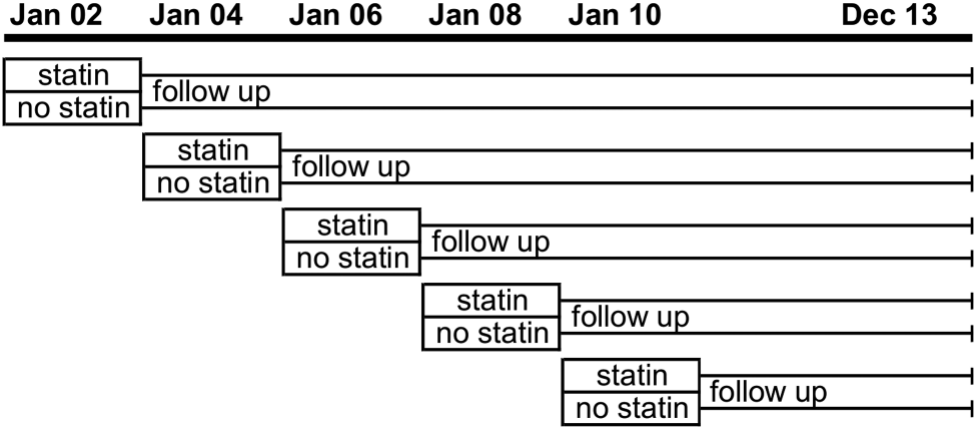
Diagrammatic representation of the staggered cohort study design.

We used THIN data to create five ‘staggered’ cohort studies with 2-year follow-up periods between 1 January 2002 and 1 January 2010 (figure 1). Further detail regarding the selection of the time window is outlined in online supplementary file S3. We assessed the effectiveness of statin prescribing using pooled data from these cohorts. Each cohort study compared statin user and non-statin user comparator groups, which were defined by whether or not an individual was prescribed a statin (one or more prescriptions) during the first 24 months of the study start. Statin users and non-users were redefined at the start of each separate staggered cohort.

10.1136/bmjopen-2016-013154.supp3 supplementary data

Statin users began follow-up on the day of their first statin prescription (the index date); for non-statin users, the index date was a randomly selected day within the 2-year exposure period. Statin users and non-users who had a CVD event recorded within 3 months of the index date were excluded because this pattern may reflect delayed recording of the CVD event, where the statin was initiated for secondary prevention of CVD. The end of follow-up was first CVD event, death or loss to follow-up (out of practice transfer or end of the study period).

The primary outcome was first MI or stroke (combined MI, haemorrhagic and ischaemic or unspecified stroke). Secondary outcomes were all-cause mortality, MI, stroke, change in total cholesterol level/concentration (for those with available records) 1 and 2 years after the index date. We selected these outcomes because other studies have identified high positive predictive values (85–95% for CVD events and 99% for all-cause mortality) for MI,^41^ ^42^ stroke^43^ ^44^ and all-cause mortality^45^ in THIN and closely related databases. We also considered investigating coronary heart disease (CHD) as an outcome, but feasibility work suggested that some CHD Read codes such as ‘unspecified CHD’ may reflect retrospective recording of pre-existing CHD.

We extracted data on covariates recorded during the 12 months before the index date (see online supplementary files S1 and S3). Covariates included age, sex, type of SMI, blood pressure, weight, height, cholesterol concentration, diabetes, smoking status, Townsend score, antihypertensive use, non-statin lipid modification (eg, fibrates), heavy alcohol drinking, familial hypercholesterolaemia, hypothyroidism, chronic kidney disease, chronic obstructive pulmonary disease, asthma, atrial fibrillation, predominant antipsychotic, mood stabilising drug, antidepressant drug, annual consultation rate and cancer diagnosis.

Unobserved covariate data (total cholesterol, height, weight, systolic blood pressure and smoking status) were estimated for the full study population using multiple imputation with chained equations to generate 10 imputed data sets.^46^ Data were separately imputed by gender in each of the five cohorts and used covariate data measured within 3 years of baseline. CVD events and Nelson-Aalen estimates of the cumulative hazard function for the time to event were included in the imputation model.^47^ A detailed description of the model and subsequent data checks is outlined in online supplementary file S3.

### Analysis

Data for each of the five cohort studies were pooled to obtain average estimates for the whole study period. Incident rate ratios (IRRs) for the association between statin prescribing and combined MI and stroke were estimated using a Poisson regression model with receipt of a statin prescription as the exposure and first MI or stroke event as the outcome: the log of follow-up time was used as an offset. Since the same individual could be included in more than one cohort, the ‘robust sandwich estimator’ was used to calculate conservative estimates of variance.^48^ The model was adjusted for covariates outlined previously. We also assessed the impact of log-transforming continuous variables and including interaction terms for age and sex.

All analyses were undertaken using an intention-to-treat approach with exposure defined by statin prescribing at the index date. The primary analysis was repeated for: all-cause mortality (death from any cause), first stroke (haemorrhagic or ischaemic stroke) and first MI to identify whether the effectiveness of statins was substantially altered for each of these outcomes. Changes in total cholesterol concentration measured during the 1 and 2 years after the index date (ie, 1–365 or 366–730 days after the index date, respectively) were evaluated for statin users and non-users in individuals with complete data at baseline. For this analysis, linear regression models with total cholesterol at 1 or 2 years after the index date were developed using the same array of covariates as for the main analysis. Change in cholesterol as an outcome for individuals who did not have complete data was not assessed. Data management and analysis were undertaken in Stata V.14.

## Results

**Figure 2:**
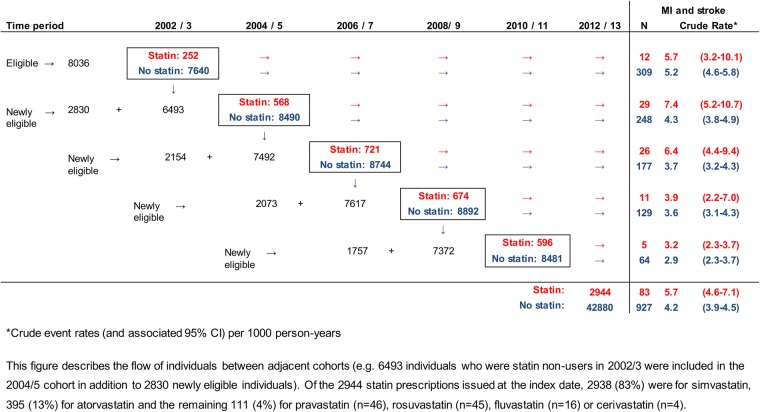
Flow chart of statin users and non-users contributing data to each of the five cohorts in the full population. MI, myocardial infarction.

Overall, 45 830 participants (defined as total statin users and non-users under follow-up) were included in the pooled data set. These participants reflect data for 16 854 individuals with schizophrenia or bipolar disorder who, on average, were included in 2.7 of the five possible cohorts. A total of 25 statin users and 27 non-statin users were excluded because they had a CVD event recorded within 3 months of the index date. A flow chart outlining numbers of statin users and non-users in and the associated event rate for each of the five cohort studies is outlined in figure 2. Table 1 describes the baseline characteristics of statin users and non-users for the full study population (using imputed data) compared with the subset with fully observed covariate data at baseline. Of note, total cholesterol concentration at baseline differed in statin users and non-users. The distribution of estimated CVD risk scores was different for statin users and non-users, with baseline risk being higher (on average) for statin users. Statin users also had greater levels of deprivation, consulted more frequently and had higher frequencies of prescribing—including antihypertensives and antidepressants—and to have familial hypercholesterolaemia (for which statin therapy is indicated; table 1).

**Table 1:** Characteristics of the study population at baseline (for complete records and the full population after imputation of unobserved data)

Characteristic at baseline	Complete cases				Full population			
	Statin non-user	IQR [or %]	Statin user	IQR [or %]	Statin non-user	IQR [or %]	Statin user	IQR [or %]
Total participants under follow-up*	**5201**		**1714**	** **	**42 886**		**2944**	
Bipolar	2612	[50%]	835	[49%]	21 883	[51%]	1460	[50%]
Median age (years)	55	(48, 64)	58	(50, 65)	54	(47, 64)	59	(51, 67)
Male	2438	[47%]	866	[51%]	19 191	[45%]	1400	[48%]
Diabetes (yes)	1156	[22%]	943	[55%]	3393	[8%]	1020	[35%]
Median systolic BP (mm Hg)†	131	(120, 141)	136	(125, 147)	130	(120, 141)	137	(126, 148)
*Per cent imputed*				* *		*[43%]*		*[7%]*
Median BMI†	28	(25, 33)	30	(26, 34)	27	(24, 31)	30	(26, 34)
*Per cent imputed*				* *		*[62%]*		*[30%]*
Median cholesterol concentration (mmol/L)†	5.3	(4.7, 6.0)	6.2	(5.5, 7.1)	5.4	(4.8, 6.2)	6.4	(5.6, 7.2)
*Per cent imputed*				* *		*[77%]*		*[10%]*
Smoker†	3004	[58%]	1120	[65%]	23 486	[55%]	1786	[61%]
*Per cent imputed*				* *		*[1%]*		*[0.1%]*
Median Framingham CVD risk score (BMI)								
Women								
40–49 years	6	(4, 10)	10	(7, 16)	5	(4, 7)	10	(6, 14)
50–59 years	11	(8, 15)	19	(12, 26)	10	(7, 14)	17	(11, 24)
60–74 years	20	(13, 30)	29	(19, 42)	17	(12, 25)	25	(17, 38)
75–84 years‡	34	(23, 49)	36	(27, 56)	26	(18, 36)	34	(24, 48)
Men								
40–49 years	14	(10, 20)	20	(15, 28)	12	(9, 16)	19	(14, 26)
50–59 years	26	(18, 35)	33	(2342)	22	(16, 30)	32	(2241)
60–74 years	41	(29, 55)	47	(35, 59)	36	(26, 47)	45	(34, 57)
75–84 years‡	58	(45, 71)	62	(48, 80)	49	(39, 63)	58	(48, 79)

**Characteristic at baseline**	**Complete cases**				**Full population**			
	**Statin non-user**	**Per cent**	**Statin user**	**Per cent**	**Statin non-user**	**Per cent**	**Statin user**	**Per cent**
Total participants under follow-up*	**5201**	** **	**1714**	** **	**42 886**	** **	**2944**	** **
Number of GP consultations during baseline								
<4	630	12%	150	9%	10 906	25%	322	11%
4–6	1024	20%	323	19%	9310	22%	568	19%
7–12	1679	32%	561	33%	11 506	27%	942	32%
>12	1868	36%	680	40%	11 158	26%	1112	38%
Antipsychotic use								
None	1926	37%	649	38%	20 061	47%	1178	40%
First generation	1091	21%	384	22%	8932	21%	630	21%
Second generation	2184	42%	681	40%	13 886	32%	1136	39%
Townsend score quintile								
1 (least deprived)	813	16%	238	14%	7280	17%	448	15%
2	823	16%	253	15%	7419	17%	462	16%
3	987	19%	336	20%	8653	20%	595	20%
4	1278	25%	380	22%	9731	23%	626	21%
5 (most deprived)	1214	23%	469	27%	9101	21%	755	26%
Not recorded	86	2%	38	2%	969	2%	58	2%
Year of cohort								
2002	268	5%	98	6%	7752	18%	285	10%
2004	592	11%	336	20%	8718	20%	605	21%
2006	1024	20%	453	26%	8901	21%	747	25%
2008	1387	27%	427	25%	8994	21%	697	24%
2010	1930	37%	400	23%	8521	20%	610	21%
Total participants under follow-up*	**5201**	** **	**1714**	** **	**42 886**	** **	**2944**	** **
Antihypertensive use (yes)	1201	23%	1065	62%	5685	13%	961	33%
Antidepressant use (yes)	2156	41%	801	47%	17 252	40%	1371	47%
Mood stabiliser use (yes)	1815	35%	538	31%	13 166	31%	922	31%
Asthma (yes)	596	11%	211	12%	4387	10%	336	11%
COPD (yes)	224	4%	85	5%	1300	3%	132	4%
Familial hypercholesterolaemia (yes)	38	0.7%	173	10%	77	0.2%	302	10%
Atrial fibrillation (yes)	59	1.0%	16	0.9%	406	1.0%	41	1.4%
Cancer diagnosis in past year (yes)	80	2%	22	1.3%	607	1.4%	39	1.3%
Hypothyroidism (yes)	47	0.9%	21	1.2%	342	0.8%	34	1.2%
Chronic kidney disease (yes)	424	8%	102	6%	1398	3%	188	6%
Heavy drinker (yes)	933	18%	293	17%	5994	14%	469	16%
Non-statin lipid modification§ (yes)	66	1.3%	34	2%	196	0.5%	51	2%

*Total participants under follow-up describes the number of statin users and non-users who were included in the pooled data sets derived from combining cohorts from different time periods.

†Denotes variables where unobserved baseline values were imputed in the full population data set.

‡Framingham risk score not validated for individuals aged >74 years.

§Refers to lipid-modifying drugs that are not statins, for example, fibrates.

BMI, body mass index; BP, blood pressure; CVD, cardiovascular disease; GP, general practice.

Modelled associations between covariates and statin prescribing or CVD events are outlined in the supplementary material for complete cases (see online supplementary file 4, table S1) and the imputed data set (see online supplementary file 4, table S2).

10.1136/bmjopen-2016-013154.supp4 supplementary data

The outputs of each of the regression models outlined in the results are included as a supplementary file (see online supplementary file 4).

The crude rate of first MI and stroke per 1000 person-years of follow-up was 5.74 (95% CI 4.62 to 7.11) in statin users and 4.17 (95% CI 3.91 to 4.45) in statin non-users in the full population (further outlined in figure 2). The crude IRR for the association between statin prescribing and MI and stroke events was 1.39 (95% CI 1.11 to 1.74) for the full study population: which was reduced to 0.89 (95% CI 0.68 to 1.15) after adjustment for all additional covariates (see online supplementary file 4, table S3). Inclusion of quadratic or log-transformed versions of continuous variables (age, total cholesterol, systolic blood pressure, body mass index) in the model did not have a marked impact on the results (data not shown).

The crude IRR for the association between statin prescribing and all-cause mortality was 1.02 (95% CI 0.90 to 1.14) and was reduced to 0.89 (95% CI 0.78 to 1.02) after adjusting for all covariates as above (see online supplementary file 4, table S3).

The crude IRR for the association between statin prescribing and first MI was 1.51 (95% CI 1.04 to 2.18) and was reduced to 0.75 (95% CI 0.48 to 1.15) after adjusting for all covariates (see online supplementary file 4, table S3).

The crude IRR for the association between statin prescribing and first stroke was 1.31 (95% CI 0.99 to 1.74) and was reduced to 0.96 (95% CI 0.68 to 1.15) after adjusting for all covariates (see online supplementary file 4, table S3).

A total of 82% and 73% of the 1714 statin users had measurements of total cholesterol recorded within the 1 and 2 years after the index date, respectively. By comparison, 55% and 57% of statin non-users had a measurement of cholesterol recorded within the 1 and 2 years after the index date, respectively.

In statin users, total cholesterol decreased on average by 1.7 mmol/L (27%) from baseline concentrations of 6.3–4.6 mmol/L at both 1 and 2 years after baseline (table 2). In contrast, among statin non-users, mean total cholesterol in each individual was slightly decreased by 0.1 mmol/L (2%) from baseline concentrations of 5.4–5.3 mmol/L at both 1 and 2 years after baseline (table 2).

**Table 2:** Summary statistics for change in total cholesterol within 1 and 2 years of the index date

Group	Description of metric	Baseline	1 year	2 years
Statin non-users	Number under follow-up	5201	2865	3002
	Mean cholesterol concentration mmol/L (SD)	5.4 (1.0)	5.3 (1.0)	5.3 (1.0)
	Change from baseline mmol/L (%)	–	**−**0.1 (**−**2%)	**−**0.1 (**−**2%)
Statin users	Number under follow-up	1714	1409	1253
	Mean cholesterol concentration mmol/L (SD)	6.3 (1.2)	4.6 (1.0)	4.6 (1.1)
	Change from baseline mmol/L (%)	–	**−**1.7 (**−**27%)	**−**1.7 (**−**27%)

The adjusted linear regression model showed a mean reduction in total cholesterol among statin users (relative to non-users) of 1.3 mmol/L (95% CI 1.2 to 1.4; see online supplementary file 4, table S4) and 1.2 mmol/L (95% CI 1.1 to 1.3; see online supplementary file 4, table S5) at 1 and 2 years after the index date, respectively. Although statin use was the most important predictor of change in cholesterol concentration at both time points, baseline cholesterol was also strongly associated with subsequent changes in total cholesterol (coefficients of −0.36 and −0.41 at 1 and 2 years, respectively; see online supplementary file 4, tables S4 and S5, respectively).

## Discussion

### Principal findings

**Figure 3:**
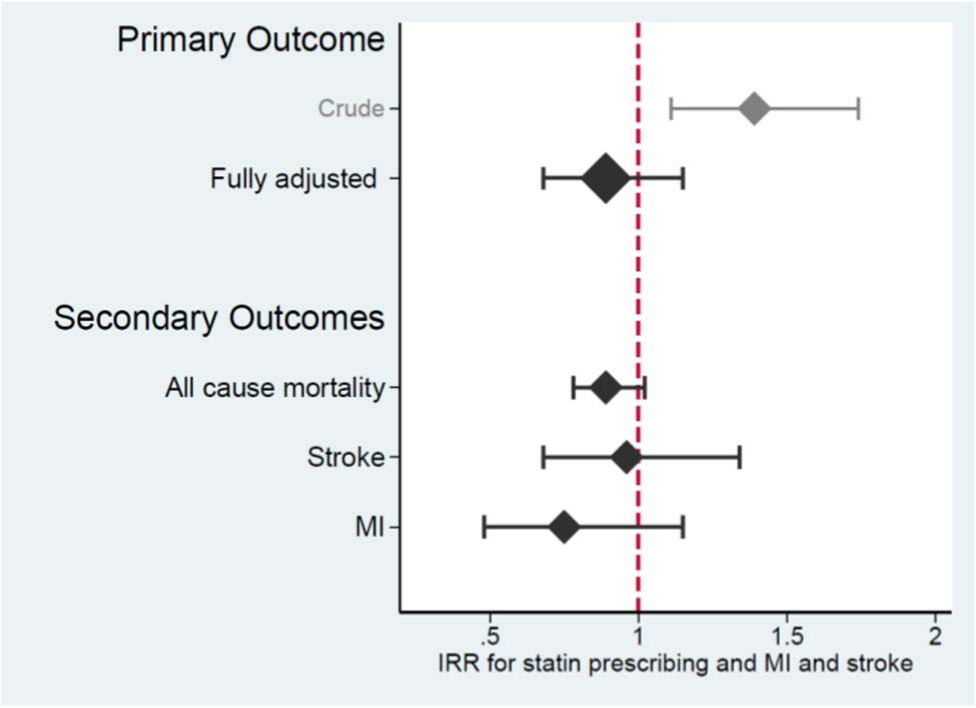
Key results for the association between statin prescribing and the rate of MI, stroke and all-cause mortality. IRR, incident rate ratio; MI, myocardial infarction.

Our results provide evidence that statin prescribing to people with SMI may carry a similar level of benefit to the general population with observed reductions for total cholesterol of 1.2 mmol/L for up to 2 years. While non-significant, the effect estimates for combined MI and stroke (IRR 0.89; 95% CI 0.68 to 1.15) and all-cause mortality (0.89 with 95% CI 0.78 to 1.02; figure 3) were broadly similar to published estimates from RCT meta-analysis^7^ and an externally validated observational study of people without SMI.^49^

### Comparison with other studies

An IRR of 0.89 (with 95% CI 0.68 to 1.15) is compatible with rates of MI and stroke being 11% lower among statin users than non-users within the study sample, but which—through chance alone—might not translate into a decreased event rate in the population. Thus, although the point estimates for our study are similar to observational studies of statins in the general population,^49^ ^50^ we cannot conclude that statins are effective in preventing CVD events in people with SMI. Sample size estimation for this type of study design is not straightforward: however, Poisson counting error (see online supplementary file S5 for more detail) provides an indication that our study was likely to be adequately powered to detect a reduction in the rate of MI and stroke with a magnitude of effect similar to that observed in statin RCTs (22% reduction)—but not observational studies (14% reduction)—in people without SMI.^7^ The estimated association between statin prescribing and all-cause mortality (IRR of 0.89) was also similar to those derived from RCT meta-analysis^7^ and observational studies,^49^ although scope for direct comparison is limited by marked variations in mortality rate and duration of follow-up between our study and RCTs included in the meta-analysis.^7^

10.1136/bmjopen-2016-013154.supp5 supplementary data

Statin use was associated with a mean decrease in total cholesterol concentration at 1 and 2 years after the index date of 1.3 and 1.2 mmol/L, respectively (equivalent to a 20–21% decrease from the index date), after adjusting for differences in the baseline characteristics of statin users and non-users. RCTs with a minimum of 6 months of follow-up in people without SMI have identified a similar net decrease in total cholesterol of 1.05 mmol/L (95% CI 0.76 to 1.35), with variation between difference types and dosage of statin.^7^ Two other studies (that included a control group) have investigated statin prescribing in people with SMI.^51^ ^52^ A non-randomised study compared changes in lipid levels during a 12-week period in 52 individuals taking 10 mg rosuvastatin daily and 48 individuals not taking statins: total cholesterol concentration was decreased by an additional 2.9 mmol/L (35%) in statin users.^51^ A pilot study randomised 60 individuals to receive 40 mg pravastatin daily or no statin for 12 weeks and reported smaller decreases in total cholesterol of 0.5 mmol/L (11%) in statin use. This pilot study also examined changes in cholesterol at 6 weeks after baseline and established that the reduction in total cholesterol was greater at 6 weeks than 12 weeks, which may indicate issues with the long-term effectiveness and/or adherence to statins.^52^

### Strengths and weaknesses

The cholesterol reduction observed in our study lies at approximately the mid-point between the two published studies outlined above, which examined rosuvastatin and pravastatin (10 and 40 mg daily dosage, respectively). This result is consistent with estimates of the cholesterol-lowering activity of statins in the general population, which suggest that 20–40 mg simvastatin (the most commonly prescribed statin in our study) has an intermediate level of activity relative to10 mg rosuvastatin and 40 mg pravastatin.^53^ Importantly, our results provide the first evidence that cholesterol concentration is reduced by over 1 mmol/L for up to 2 years, which suggests that medication adherence is sustained. This finding is important because it suggests—in agreement with other studies^54^ ^55^—that statin use can result in substantial reductions in cholesterol among populations with high antipsychotic usage. Furthermore, other studies in the general population have shown that the impact of statin prescribing on CVD events increases with extended use: reductions of 0.6 mmol/L of total cholesterol are associated with a 7% decrease in ischaemic heart disease during the first 2 years of therapy and >20% decreases thereafter.^56^ The decreased total cholesterol concentrations attributed to statin therapy in people with SMI may therefore translate into long-term clinically meaningful reductions in CVD.

A limitation of our study findings for total cholesterol is that these apply only to individuals who had complete data at baseline and who had one or more measurements of total cholesterol recorded in the 2 years after the index date. Although the ascertainment of cholesterol outcome data was good for statin users (73–82%), it is possible that individuals who are more compliant may be more likely to have a blood test. Therefore, our results may overestimate the potential effect of initiation of statin treatment. In addition, outcome data were less available for statin non-users (55–57%). This is potentially important because, while many trials of statins suggest that cholesterol concentration is unlikely to change substantially in the control arm, long-term temporal trends in dyslipidaemia have been observed, even among those without treatment for lipid modification.^57^

The use of observational data for studies that compare treated and untreated arms is likely to result in comparison groups that differ, especially in terms of their risk for the condition being medicated. Although this study employed strategies to increase the comparability of statin user and non-user groups, the profiles of statin users and non-users differed at baseline, with statin users having greater CVD risk and a raised crude MI and stroke event rate. These baseline differences were anticipated and—as with other studies^38^ ^40^ ^50^—addressed through use of staggered cohort design and adjustment for known confounders of the association between prescribing and CVD outcomes. The impact of these statistical adjustments was large and reversed the direction of association between statin prescribing and CVD events, thus highlighting the importance of confounding and the extent to which it may be controlled. However, this approach can only reduce bias arising from differences in measured confounders. Residual confounding arising both from time-varying changes in measured covariates (such as blood pressure within the baseline period) and unmeasured factors such as the severity of mental illness may still impact on the study results. In addition, although the outcomes investigated by this study have high positive predictive values, there is likely to be a small proportion (eg, ∼5%^41^) of events that are imperfectly recorded and may therefore reduce the accuracy of our results.

The results of statin effectiveness on CVD outcomes described by this study have focused on data for the full population with imputed values of covariate data (on blood pressure, total cholesterol, smoking status, weight and height) where these measurements were unobserved at baseline. Individuals who were not prescribed a statin but who had complete baseline data made up only a small proportion (12%) of total statin non-users included in the full analysis. It was therefore important to extend the analysis beyond individuals for whom complete data were available. We made every attempt to develop a robust approach towards imputing missing covariate data (further outlined in supplementary files) but acknowledge that our estimates are not a substitute for fully observed data. In particular, it was not possible to develop an imputation model that included high-density lipoprotein cholesterol (HDL-C) because >90% of data were missing for HDL-C in early time periods in the study. Lipid profile (including HDL-C fraction and trigylcerides) is both predictive of CVD events and associated with SMI, through mechanisms such as poorer diet, which is associated with a lower HDL-C fraction.^58^ Incomplete characterisation of lipid profile within this study may result in some residual confounding, thereby potentially biasing our estimate of the effectiveness of statin prescribing, probably towards the null. The high level of similarity between results for the primary outcome using imputed data relative to complete cases (outlined in online supplementary file S4) adds greater certainty to our findings.

### Meaning

The results from this study provide evidence that the potential impact of statin prescribing on intermediate outcomes in people with SMI has a magnitude that is similar to the general population. We identified statistically significant reductions in total cholesterol (of 1.2 mmol/L up to 2 years, p<0.001), suggesting that medication adherence in people with SMI is sufficient to support effective lipid modification. Both CVD screening and statin prescribing should be encouraged as a potential means of decreasing the mortality gap in SMI. However, the impact of these interventions should be further evaluated relative to other interventions—such as smoking cessation—for primary prevention of CVD.
