# Supplementary material for: Statin prescribing for people with severe mental illnesses: a staggered cohort study of ‘real-world’ impacts
Source: BMJ Open. 2017 Mar 7;7(3):e013154. doi: 10.1136/bmjopen-2016-013154 (PMC5353294; doi:10.1136/bmjopen-2016-013154)
Supplement: supplementary data [file bmjopen-2016-013154supp1.pdf]

Case definitions

Read code and drug code lists were compiled using standard methods (Dave and Petersen, 2009). Full code lists for all case definitions are available on request from the authors.

SMI diagnoses

Schizophrenia: *schizophrenia, schizoaffective, paranoid schizophrenia*

Bipolar disorder: *bipolar affective, psychotic depression, hypomanic psychoses, manic depressive, manic disorder, manic psychoses*

Individuals with other types of psychosis, or who only had a record indicating that they were on the SMI register, were not included in the study.

Other SMI: *non-organic psychoses, oneirophrenia, affective psychoses, depressive psychoses, paranoid states, reactive psychosis*

SMI register: *severe mental illness register, national service framework mental health*

CVD events

CVD events were identified from Read codes held in the medical records: CVD events were classified according to whether the Read code described a new (incident) or pre-existing (prevalent) condition as well as the type of condition (myocardial infarction (MI), unstable angina, angina, haemorrhagic stroke, ischaemic and unspecified stroke, transient ischaemic attack (TIA), surgery, unspecified coronary heart disease (CHD) or unspecified CVD). Records of death that were associated with a CVD diagnosis were identified from Read codes held in the cause of death field (*1016500000*) in the additional health records. In addition, Read codes for CVD that were recorded after the date of death were assumed to reflect fatal CVD events.

Terms for identifying CVD included:

MI: *myocardial infarct, MI, myocardial infarction, myocardial ischaemia, acute Q-wave infarct, acute non-Q wave infarct, anteroseptal infection, anterolateral infection, anteropical infraction, acute papillary muscle infarction, acute atrial infarction, subendocardial infarction, Dressler's syndrome, ECG* AND *infarction*, *acute ischaemic heart disease, heart attack, post infarction pericarditis, thrombosis – coronary, coronary thrombosis, mural thrombosis*

Unstable angina and acute coronary syndrome: *coronary thrombosis not resulting in myocardial infarction, ACS, stenocardia, unstable angina, crescendo angina, subendocardial ischaemia, coronary artery spasm, preinfarction syndrome, impending infarction, refractory angina, worsening angina, acute coronary syndrome, acute coronary insufficiency, acute and subacute ischaemic heart disease, microinfarction of heart, angina at rest, transient myocardial infarction*

Angina: *stable angina, angina pectoric, angina on effort, Prinzmetal's angina, angina control, syncope angina, chronic ischaemic heart disease, nocturnal angina, angina decubitus, status anginosus, new onset angina, angina, chronic coronary insufficiency, chronic myocardial ischaemia, post infarct angina*

Haemorrhagic stroke: *bulbar haemorrhage, intracerebral haemorrhage, capsule haemorrhage, cerebellar haemorrhage, cortical haemorrhage, non-traumatic intracranial haemorrhage, pontine haemorrhage, basal nucleus haemorrhage*

Ischaemic and unspecified stroke*:* *unspecified occlusion or stenosis of cerebral arteries, cerebral infarction, lateral medullary syndrome, stroke, basilar artery occlusion, brainstem infarction, Wallenberg syndrome, carotid artery occlusion, carotid artery syndrome, precerebral artery occlusion, vertebral artery occlusion, cerebral embolism, cerebral artery syndrome, infarction of basal ganglia, pure sensory lacunar syndrome, right sided CVD, left sided CVD amaurosis fugax, subclavian steal syndrome*

TIA: *transient cerebral ischaemia, transient ischaemic attack, TIA, intermittent cerebral ischamia*

Surgery: *angioplasty, operations on coronary artery, replacement* AND *coronary artery*, *bypass* AND *coronary artery*, *connection* AND *coronary artery*, *exploration of coronary artery, repair of coronary artery, stent* AND *coronary*, *anastomosis* AND *coronary, Endarterectomy* AND *coronary, percut transluminal coronary thrombolysis,*

Unspecified CHD: *ischaemic, coronary vessel disease, coronary artery disease, atherosclerotic heart disease, atherosclerotic cardiovascular disease, CHD*

Unspecified CVD: *cerebrovascular disease, cerebral artery syndrome, cardiovascular disease, stenosis of precerebral arteries, vertebrobasilar insufficiency, basilar artery syndrome, CVD, cerebral atherosclerosis, cerebral ischaemia*

Statin prescriptions

Statin prescriptions were identified from therapy records corresponding to drug codes for statins outlined in Chapter 2.12 (lipid-modification) of the BNF (Joint Formulary Committee, 2012) and by searching for generic drug names including the term “statin”. Terms for identifying statin medication included:

Statin: **statin, fluvastatin, simvastatin, rosuvastatin, cerivstatin, atorvastatin, pravastatin, lipitor, lescol, lipostat, crestor, zocor*

Confounders

Age in years: calculated as (date of interest minus the date of birth (held in adult patient records as *year of birth*)) divided by 365.25

Gender: held in the patient records (*sex*)

Smoking status: Read codes held in either the additional health records (*1003040000*) or the medical records were used as follows to classify an individual’s smoking status for a given record:

> **Current smokers**: *1372.00, 1373.00, 1374.00, 1375.00, 1376.00, 137..11, 8CAg.00, 8CAL.00, E251.00, E251000, E251100, E251z00, 137H.00, 137J.00, 137P.00, 137P.11, 137Q.00, 137R.00, 137V.00, 1372.11, 137b.00, 137C.00, 137c.00, 137d.00, 137f.00, 137g.00, 137G.00, 137h.00, 137M.00, 8H7i.00, 8HTK.00, 9N2k.00, 9N4M.00, 67A3.00, ZG23300, ZV4K000, 8I2I.00, 8I39.00, ZRBm200, ZRBm211, 137Y.00, 6791.00*
>
> **Ex-smokers:** *1377.00, 1378.00, 1379.00, 137A.00, 137B.00, 137F.00, 137K.00, 137N.00, 137O.00, 137S.00, 137T.00, 13p4.00*
>
> **Never smokers:** *1371.00, 1371.11*
>
> **Smoking cessation:** Prescriptions recorded in the therapy records were used to indicate smokers attempting to quit (i.e. transition from current to ex-smoker status) and included the following generic drug names: *nicotine, varenicline, menthyl valerate and quinine, buproprion.*

Weight: Records of weight (and the associated unit of measurement, e.g. kilograms) were identified from the additional health records (*1005010200*)

Height: Records of height (and the associated unit of measurement, e.g. centimetres) were identified from the additional health records (*1005010100*) and retained for measurements taken at or after the age of 21 years

BMI: BMI was estimated as weight in kilograms divided by height in metres squared

Diabetes: Records indicating diabetes were identified using additional health records (*1009100000, 1020000004, 1001400327, 1001400140 and 1009120000*)**,** which indicate checks in people with a diagnosis of diabetes (e.g. for diabetic retinopathy) or diabetes medication. Read codes held in the medical records indicating the following conditions were also used to identify people with diabetes.

Diabetes: *diabetic, diabetes, insulin, referral to diabetologist, diabetology, IDDM, NIDDM,*

In addition Read codes that started with *C1* (endocrine, nutrition, metabolic and immunity disorders) were reviewed for relevant terms that were missed by the initial word search. Code lists were reviewed by a clinician for irrelevant codes, which were excluded.

Systolic and diastolic blood pressure: Records of blood pressure (and the associated unit of measurement; mmHg) were identified from the additional health records (*1005010500*).

Antihypertensive medication: Records indicating treatment with antihypertensive medication was identified as drugs listed in Chapter 2.2.1 (thiazides and related diuretics), 2.5.5.1 (angiotensin-converting enzyme inhibitors), 2.5.5.1 (angiotensin-II receptor agonists), 2.6.2 (calcium-channel blockers) and 2.4 (beta-adrenoceptor blocking drugs) of the BNF (Joint Formulary Committee, 2012).

Total cholesterol concentration and HDL-C: Records of cholesterol concentration (and the associated unit of measurement) were identified from the additional health records (total cholesterol; *1001400017* and HDL-C; *1001400031*).

Psychiatric medication

Individuals who were prescribed antipsychotics, antidepressants or mood stabilizers were identified using code lists for the following generic drug names:

Antipsychotics

First generation: *benperidol, chlorpromazine, chlorprothixene, droperidol, flupentixol, fluphenazine, fluspirilene, haloperidol, levomepromazine, loxapine,*

pericyazine, perphenazine, pimozide, pipotiazine, promazine, thiopropazate, thioproperazine, thioridazine, trifluoperazine, trifluperidol, zuclopenthixol

Second generation: *amisulpride, aripiprazole, asenapine, clozapine, olanzapine, oxypertine, paliperidone, quetiapine, remoxipride, risperidone, sertindole, sulpiride, zotepine*

Mood-stabilisers

carbmazapine, lamotrigine, lithium, sodium valproate

Antidepressants

Monoamine-oxidase inhibitor: *iproniazid, isocarboxazid, moclobemide, phenelzine, tranylcypromine, trifluoperazine*

Selective serotonin re-uptake inhibitor (SSRI): *citalopram, escitalopram, fluoxetine, fluvoxamine, paroxetine, sertraline*

Tricyclic: *amitriptyline, amoxapine, bolvidon, butriptyline, clomipramine, desipramine, dosulepin, dothiepin, doxepin, imipramine, iprindole, lofepramine, maprotiline, merital, mianserin, nomifensine, nortriptyline, perphenazine, protriptyline, sinequan, tofranil, trazodone, trimipramine, viloxazine, zimelidine*

Other: *agomelatine, duloxetine, flupentixol, l-tryptophan, mirtazapine, nefazodonem, reboxetine, tryptophan, venlafaxine*

Healthcare Factors

Annual face-to-face consultation rate in primary care was used as a measure of healthcare utilisation, types of consultation included consultations both within surgery opening hours and out of hours. Face to face consultations were identified from the following consultation codes: nursing home visits were not included.

Face to face consultations: *walk-in centre (consultation code 034), night visit, practice (006), emergency consultation (018), clinic (001), community clinic (100), surgery consultation (009), acute visit (011), out of hours, non-practice (008), home visit (027), out of hours, practice (007), night visit (110), co-op surgery consultation (036), co-op home visit (037), follow-up/routine visit (003)*

Hypothyroidism: *hypothyroidism, cretinism, Pendred's syndrome, Goitrous cretin, myxoedema, thyroid deficiency, thyroid insufficiency, acquired atrophy of thyroid*

Cancer (broad description of diagnosis type – please contact the authors for a detailed break-down): *neoplasm (not otherwise specified), dermatological, gastro-intestinal, metastasis, haematological, chest, respiratory, bone, central nervous system*

COPD: *COPD, chronic obstructive pulmonary disease, chronic obstructive airways disease, chronic bronchitis, bronchiolitis obliterans, emphysema, Sawyer-Jones syndrome*

Asthma: *asthma, hyper-reactive airways disease, status asthmaticus, allergic bronchitis*

CKD: *chronic kidney disease (stages 3-5), CKD (stages 3-5), chronic renal disease, chronic renal impairment*

Atrial fibrillation: *atrial fibrillation*

Heavy drinking: review of additional health records (*1003050000*) and the associated units for drinking habits indicating consumption of ≥28 (women) and 35 (men) units per week. In addition, the following Read codes were used to identify heavy drinkers.

> **Heavy drinkers:** *alcohol intake above recommended sensible limits, binge drinker, moderate drinker, non-dependent alcohol abuse, alcoholic hepatitis, chronic hepatitis, hepatic failure, liver failure, alcohol amnestic syndrome, alcohol dependence, alcohol problem drinking, alcohol withdrawal, alcohol-induced chronic pancreatitis, alcoholic dementia, alcohol sclerosis of liver, alcoholics anonymous, alcoholic psychoses, portal cirrhosis, chronic alcoholic syndrome, harmful alcohol use, heavy drinker, hepatic failure, Laennec's cirrhosis, recurrent hepatitis, toxic hepatitis, toxic liver, Wernicke's,*

Read codes indicating "history of alcoholism" or "ex-alcoholic" were not included.

Familial hypercholesterolaemia: Read codes indicating *familial hypercholesterolaemia* and/or a record of total cholesterol concentration >7.5mmol/L was used to identify people with possible familial hypercholesterolaemia (National Institute for Health and Clinical Excellence, 2008a).

Non-statin lipid modification: review of therapy records for all drugs outlined in Chapter 2.12 of the BNF (Joint Formulary Committee, 2012) excluding all drugs previously identified as statins

<u>Exclusion criteria</u>

Terminal illness was defined as Read codes held in medical records relating to the following conditions: *terminal illness – late stage, last days of life, terminal care, palliative care, GSF supportive care, Liverpool care pathway, end of life advanced care plan, terminal care, end of life care,*

Raised liver enzymes was defined as: 3x the upper limit of the reference ranges for either alanine transaminase (ALT) or aspartate aminotransferase (AST) as indicated in addition health records (*1001400006* and 1001400007, for ALT and AST respectively). For ALT and AST the current upper limits of the reference ranges for NHS Camden are 33 and 31 IU/L, respectively. These values were inflated by 25% and multiplied by 3 to give highly conservative values of 124 (ALT) and 116 IU/L (AST) for exclusion. These individuals were excluded because statins are contraindicated for people with elevated liver enzymes (Joint Formulary Committee, 2012).

Dementia was defined as Read codes held in medical records relating to the following conditions: *Alzheimer's, dementia, other senile and presenile organic psychoses, presbyophrenic psychosis, senile psychosis,*
