# Supplementary material for: Statin prescribing for people with severe mental illnesses: a staggered cohort study of ‘real-world’ impacts
Source: BMJ Open. 2017 Mar 7;7(3):e013154. doi: 10.1136/bmjopen-2016-013154 (PMC5353294; doi:10.1136/bmjopen-2016-013154)
Supplement: supplementary data [file bmjopen-2016-013154supp2.pdf]

Estimating the effectiveness of statins for the primary prevention of cardiovascular disease in patients with severe mental illness

Rationale for estimating the effectiveness of statins in people with SMI

People with a severe mental illness (SMI) such as schizophrenia or bipolar disorder are more likely to develop cardiovascular disease (CVD) and experience worse outcomes following a cardiovascular event than the general population.[1;2] CVD (including heart disease, myocardial infarction and stroke) is the leading cause of death amongst people with SMI and drives much of the 13-30 year deficit in life-expectancy relative to the general population.[2;3]

The increased risk of CVD is attributed to risk factors such as smoking, poor diet and physical inactivity that are more prevalent among people with SMI than the general population.[4] CVD risk may also be increased by some antipsychotic agents such as clozapine, which are associated with weight gain, increased blood lipid concentration (dyslipidaemia) and elevated risk of developing type II diabetes.[5-7] Furthermore, there is evidence that physical health awareness amongst people with SMI and those caring for them is poor, and that preventative CVD care may be undersupplied or delayed.[8-10] Taken together, these findings highlight the need for improved primary prevention of CVD in individuals with SMI.

Statins lower blood cholesterol concentration by inhibiting cholesterol synthesis and promoting the removal of low density lipoprotein cholesterol from blood. Randomised controlled trials (RCTs) have shown statins to be cost-effective for managing dyslipidaemia and preventing CVD events in high risk people (recently defined for the UK as ≥10% risk of CVD over a ten year period, but historically 20%).[11-19] However, people with SMI are under-represented in statins trials and the effectiveness of statins may differ relative to the general and trial populations due to increased CVD risk, lower medication adherence and antipsychotic exposure.

Direct comparison of the SMI and statins trials populations has not been undertaken: however, the relative risk of CVD events amongst people with SMI is approximately twice that of people without SMI.[1] Mental illness is generally associated with poorer medication adherence, although estimates specifically for statin medication are not known for individuals with SMI.[20-22] Additionally, some antipsychotic agents interact with sterol regulatory binding elements (which control lipid synthesis) resulting in increased cholesterol concentration and may therefore counteract the cholesterol-lowering action of statins.[23-25] Furthermore, several large statins trials have explicitly excluded participants with psychological conditions[14;26;27] or excluded individuals perceived as less likely be compliant with treatment.[28-31]

To date, no RCTs have examined the effectiveness of statins in people with SMI. Only one study has attempted to estimate the effectiveness of statins for people with SMI relative to statin untreated comparators: the results provide some assurance that statins can deliver clinically meaningful reductions in cholesterol in individuals with SMI.[32] However, the strength of evidence is limited by sample size (n=100), short duration of follow up (3 months), non-randomised design and type of statin (rosuvastatin, which is priced 10-20 times higher than other statins used for primary prevention).[33]

Following the introduction of annual physical health checks for people with SMI in 2004, statins for the prevention of CVD are more frequently prescribed to people with SMI than comparable individuals without SMI (work detailed in our SRC protocol ref 13-022). Whilst the increased recognition of

cardiovascular risk amongst people with SMI is reassuring, the lack of evidence underpinning the effectiveness of statins in this patient group is concerning and warrants investigation.

Rationale for selecting our proposed study design

Our proposed study design is outlined in full in the methods section: in brief, we will develop series of cohort studies that are initiated at staggered six-monthly intervals (**Figure 1**). Within each of these studies we will compare the outcomes of individuals who initiate statin therapy within a six-month baseline period with those who do not initiate therapy, and will adjust for covariates recorded during the baseline period. The methodological features of our proposed study design have been extensively piloted in studies examining the impact of statin prescribing in primary care,[34-38] but may be unfamiliar to many researchers: we therefore outline the motivation for our choice.

To select a suitable design we appraised several possible designs[36;39;40] and assessed the extent to which these:

i) are compatible with partially observed covariate data
ii) reduce confounding by indication

Using multiple imputation to resolve issues of missing data

Cholesterol, weight, blood pressure and smoking status are important predictors of cardiovascular risk that change with time: incorporating these variables into our analyses is therefore essential for obtaining unbiased results. However, these indicators are usually only measured when clinically indicated, resulting in missing data. A range of methods are available for handling missing data including complete case analysis or other *ad hoc* missing data methods. However, when correctly implemented, multiple imputation is superior because it becomes possible to analyse data for all individuals and provides more accurate measures of the uncertainty around the effect estimate.[41] There is a growing selection of tools to help impute missing data in longitudinal datasets, including the MI suite of commands and twofold FCS specification algorithm,[42;43] which has been developed and tested using cardiovascular disease covariate data.

Multiple imputation results in many (typically 5-10) copies of the imputed dataset and can be challenging to combine with some types of methodology (such as propensity scores). This is because the imputed datasets comprise different estimates of the true unobserved value, and it therefore becomes difficult to assign a single value to each individual.[44;45] By contrast, methods for undertaking regression analysis with multiple imputed datasets are well established and feasible using standard statistical software packages.

Reducing confounding by indication

Obtaining robust estimates of the effectiveness using observational datasets is challenging due to confounding by indication. Confounding by indication describes the imbalance in characteristics between exposed and unexposed individuals, which arises in observational datasets because treatment allocation (such as a statin prescribing) is clinically indicated and not random. Treatment is therefore related to the risk of future health outcomes, such that direct comparison of exposure groups yields a biased result. Bias may also arise when the timing of exposure or start of follow up is not equitable across comparison groups: particularly when investigating treated and untreated groups because it may be unclear how the start of follow up should be defined for unexposed individuals.

Adjusting for imbalances in measured covariates at baseline

We considered use of a propensity score (a variable indicating an individual's likelihood of being prescribed a drug given their covariate data) to remove measured confounding.[46] Smeeth and colleagues used a propensity score in combination with eligibility and matching criteria to analyse observational data and obtained similar results to an RCT (The Heart Protection Study[14]).[39] However, we are unable to replicate these methods because the number of individuals with SMI is not sufficiently large to support matching statin users and non-users on similar criteria (GP practice, 5 year age band, gender, compatible time of registration). We therefore considered using the propensity score as the sole criterion for matching, but rejected this approach because a fully observed dataset is required. Traditional regression provides a good alternative to a propensity score because it is readily compatible with multiple imputation and produce similar results to a propensity score.[47] Furthermore, specific estimates for the effectiveness of statins (versus no treatment) for primary prevention of coronary heart disease found almost identical hazard ratios and confidence intervals derived from either traditional regression or propensity score methods (0.89 (0.73-1.09) and 0.88 (0.72-1.08), respectively).[36]

Statin prescribing as a time varying exposure

Defining an index date for the unexposed group remains a challenge in studies with a no treatment arm and is particularly important if the index date defines a baseline period in which time varying covariates are measured (e.g. cholesterol measured in the six months prior to the index date). In their study, Hippisley-Cox and Coupland defined the date of entry for each individual as either the earliest start date (the latest of: January 2002, registration plus 12 months, 30th birthday) for non-statin users and at first statin prescription after the earliest start date for statin users.[40] However, this leaves the study open to immortal time bias arising from the relatively earlier entry into the study that occurs for unexposed individuals. A further pitfall is using an overly long baseline period to identify exposed individuals. In the case of the Hippisley-Cox study, the entire follow up period (until December 2008; 6.5 years) is used to classify individuals as statin exposed or unexposed, which is likely to result in comparison groups that are substantially different to each other.

An additional consideration is that guidelines and patterns of statin prescribing have changed over time: it is therefore desirable to select exposed and unexposed individuals at similar time points. An added complication is that the bulk of prescribing occurs towards the end of the study period and many individuals who are initially unexposed are later prescribed a statin, such that statin exposure varies with time. Danaei and colleagues have developed a method that mimics recruitment into a series of trials: exposure is therefore defined at multiple time points, such that an individual's exposure status varies over time.[36] The use of short exposure periods (e.g. one[37] or six[36] months) also makes the selection of a plausible index dates for unexposed individuals straightforward (e.g. exposure period start or a randomly selected date within the exposure period) and should produce equivalent start dates for exposed and unexposed individuals to enter the study.

Purpose

This study aims to estimate the effectiveness of statins in reducing CVD events in people with SMI, such that these estimates are as close as possible to the results that would have been obtained through an RCT.

Objectives:

1) To compare individuals with SMI who did (exposed), or did not (unexposed), receive a statin prescription to estimate the effect of statin prescribing on primary CVD events, which are analysed according to:

I. exposure status at the index date (analogous to an intention to treat analysis)
II. exposure status at the index date as well as any subsequent changes in exposure status (analogous to a per protocol analysis)

2) Calculate effect estimates for the association between statin prescribing and:

I. all-cause mortality
II. combined fatal and non-fatal coronary heart disease events (including separate reporting for myocardial infarction)
III. combined fatal and non-fatal stroke events
IV. cholesterol concentration

Data Source

The Health Improvement Network (THIN) is a primary care database populated by data arising from GP consultations in over 500 UK practices. THIN data provide information on approximately 6% of UK residents and are representative of the whole population. THIN captures information on; patient demographics (age, sex, ethnicity and Townsend score for deprivation), morbidity (diagnoses), treatment (prescriptions including dose, quantity and dates), lifestyle and health indicators (smoking status, exercise, alcohol intake, weight and height) and consultation dates (including referral). The average follow up time for each patient is approximately 6 years. The reliability of THIN data has been tested by validation against experimental and observational evidence from the literature and found to be robust.[48;49]

Figure 1) Diagrammatic representation of the staggered multi-cohort study design. The enlarged section (bottom right) outlines addition detail such as the index dates for two hypothetical individuals in each arm of the study beginning 1st January 2002.

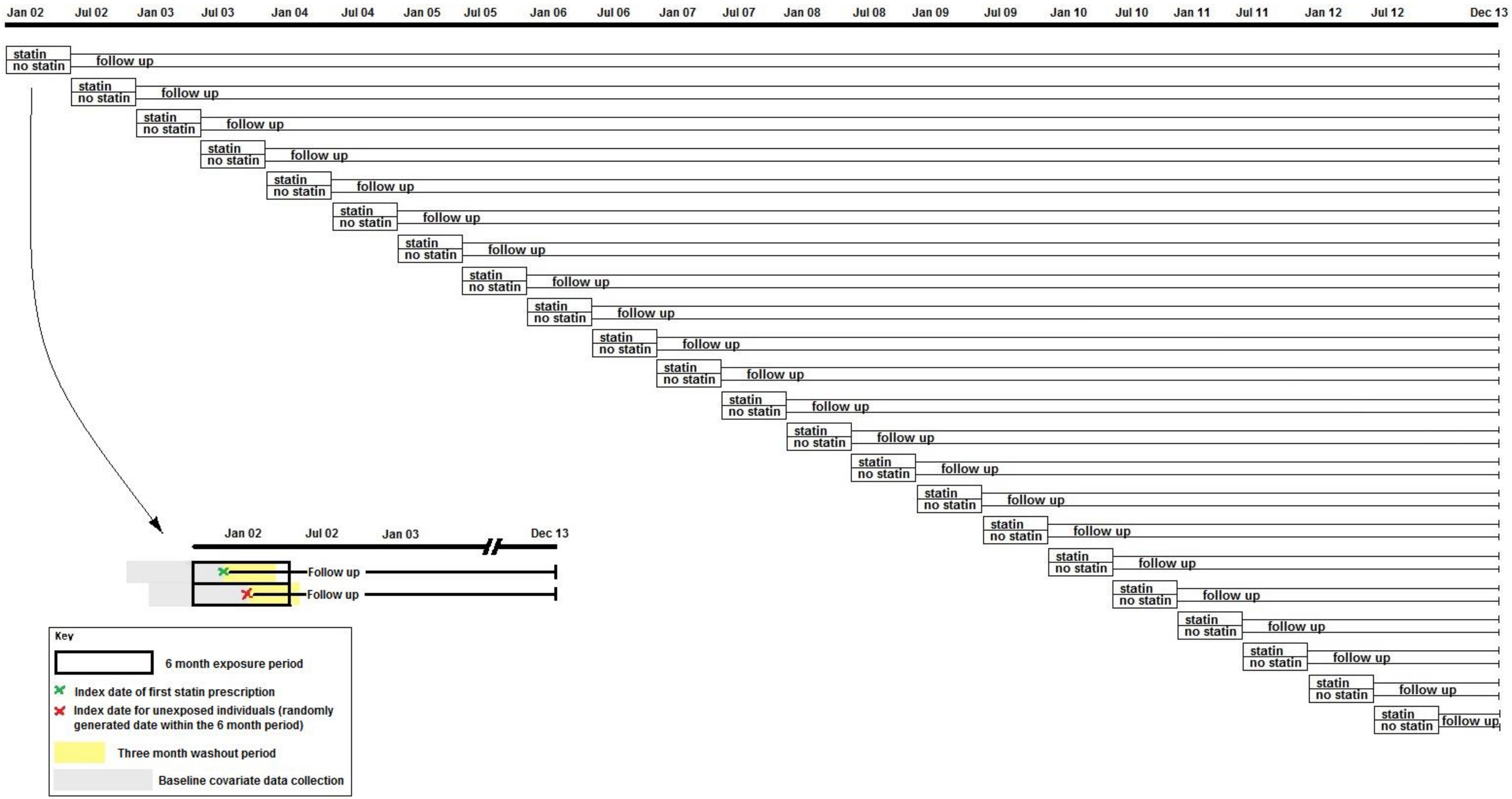

Methods

Population

23,000 individuals with schizophrenia or bipolar disorder who:

1) Are permanently registered patients
2) Consulted their GP during the study period
3) Are aged 30-84 years at baseline
4) Do not have a prior diagnosis of CVD
5) Do not have a statin prescription within the previous 24 months
6) Are registered for a minimum of six months prior to enrolment

Individuals with a diagnosis of CVD at or before the start of each study will be excluded. Entry into each study will be restricted to individuals with **no prior statin prescription in the 24 months before the study start date.** Individuals will be eligible to enter each study on or after the latest of; i) 30th birthday ii) date of registration plus 6 months, iii) date of practice acceptable mortality rate or acceptable computer usage[50].

We will exclude individuals with baseline terminal illness or a statin contraindicating condition (active liver disease: exclusion threshold values of 124 and 116 IU/L for aspartate aminotransferase (AST) and alanine transaminase (ALT), respectively) will be excluded.

Intervention and Comparison groups

The exposure status of each individual will be determined by whether or not they received a statin prescription within the six months (exposure period) following the study start.

Outcome

The primary outcome will be CVD events, with narrower measures (e.g. fatal versus non-fatal events, and division of coronary heart disease and stroke) to be explored in additional sensitivity analyses.

We will apply a similar method to Danaei[36] to evaluate the effect of statin prescribing to individuals with SMI by running 22 staggered cohort studies that are initiated every six months (**Figure 1**). The first study will start on 1st January 2002 and the last study will start on 1st July 2012. Individuals who initiate a statin within the six month exposure period will begin follow up on the day of their first statin prescription (the index date). An index date within the six month exposure period will be randomly selected for Individuals who are not exposed. In every study, each individual is followed up until the earliest of: CVD event, censoring (out of practice transfer or death) or 31st December 2013 (which marks the end of the study period and allows a one year period after the last exposure date to capture events).

A washout period (see enlarged section of **Figure 1**) will be applied to the three months following the index date. This is primarily to exclude individuals for whom statins were prescribed as secondary prevention: i.e. prescriptions after a CVD event, but where the CVD date may be incorrect in the patient's records such that the event appears to occur immediately after the index date. This scenario may arise when a GP is notified of the CVD event via a discharge letter from secondary services, or at the point at which the patient obtains a statin prescription. The washout period will be applied to both exposed and unexposed individuals in order to avoid differential biases.

To obtain an overall estimate of effect we will pool the individual effect estimates (and the associated standard errors) for each study. As individuals can be included in more than one study it will be necessary to combine the effect estimates using appropriate methods (such as using robust standard errors) that account for clustering by individual. In the main analysis an individual's exposure status will be classified in line with intention to treat, such that individuals are analysed according to their initial exposure status regardless of any changes in exposure status (e.g. initiating a statin after the exposure period, or prescriptions stop). An additional sensitivity analysis will be undertaken to evaluate the impact of statin adherence, using gaps in statin prescriptions to guide estimates of adherence.

Study Variables

1) **Presence of SMI** (in line with previous studies[1;51]): Read codes for bipolar disorder and schizophrenia,
2) **Exposure to statins:** drug code list from BNF chapter listings (statins; 2.12)
3) **Cardiovascular events:** Read codes for myocardial infarction, angina, ischaemic or unspecified stroke, haemorrhagic stroke, transient ischaemic attack, cardiovascular surgery, unspecified CVD or CHD
4) **Contraindications for statins:** including active liver disease
8) **Covariates:** on established CVD and SMI risk factors informed by published literature.

- Age
- Gender
- Townsend score
- Diabetes diagnosis or treatment
- Systolic blood pressure
- Diastolic blood pressure
- Total cholesterol concentration
- High density lipoprotein cholesterol concentration
- Weight / BMI
- Smoking status
- GP consultation rate
- Record of excessive drinking (Y/N)
- Other non-statin lipid modification drugs
- Hypertension diagnosis or treatment
- Familial hypercholesterolaemia
- Renal disease
- Hypothyroidism
- Predominant antipsychotic type
- Antidepressant use
- Chronic obstructive pulmonary disease
- Dementia

Time-varying covariate data on each individual at baseline will be obtained from records for the twelve months prior to the index date (see enlarged section of **Figure 1**). As these data are not fully observed we will use multiple imputation to estimate plausible values.

Multiple imputation

Missing covariate data in the twelve months prior to the index date will be estimated using multiple imputation. The imputation models will be separately executed for each of the 22 study datasets. The imputation model will draw upon time varying data from up to three years before and after the index date. *A priori* variables in the imputation model are: age (in 5 year bands), exposure status, diabetes status, blood pressure, cholesterol concentration, height, weight, smoking status, CVD events and the associated Nelson-Aalen cumulative hazard function estimate[52]. We will also include all other variables that will be used in the final analysis model.

Analysis

Objective 1I) To compare individuals with SMI who did (exposed), or did not (unexposed), receive a statin prescription to estimate the effect of statin prescribing on primary CVD events analysed according to exposure status at the index date

For all sections of this analysis individuals will be analysed according to their exposure status during the initial six month exposure period at the start of each cohort study. As missing data within each dataset has been estimated using multiple imputation we will use the MI suite of commands in Stata to calculate effect estimates. These commands are able to appropriately combine and calculate point estimates and confidence intervals from multiple imputed datasets.

Descriptive analyses

An initial descriptive analysis will be undertaken to describe the characteristics (including SMI condition, age, gender, diabetes status, blood pressure, body mass index, cholesterol concentration, smoking status and estimated Framingham CVD risk score[53]) of individuals in each of the studies and present a summary of these data by exposure status.

Crude and adjusted analysis of individual cohort study datasets

We will then analyse each dataset using Cox regression with statin as the exposure and CVD events as the outcome. In addition to calculating the crude hazard ratio for each study, we will adjust the Cox model for a panel of *a priori* confounders (SMI condition, age, gender, diabetes status, blood pressure, body mass index, cholesterol concentration and

smoking status) to estimate the adjusted hazard ratio and associated 95% confidence interval. We will also explore the impact of adding additional confounders (including baseline: GP consultation rate, excessive drinking, non-statin lipid modification, hypertension diagnosis or treatment, renal disease, thyroid disease, predominant antipsychotic use, antidepressant use and dementia) which were included in the multiple imputation model.

Combining the effect estimates

We will present the effect estimates yielded from each study as a Forest plot. We will then calculate the overall hazard ratio and confidence interval across all the studies using a random effects meta-analysis.[54]

Objective 1II) To compare individuals with SMI who did (exposed), or did not (unexposed), receive a statin prescription to estimate the effect of statin prescribing on primary CVD events analysed according to exposure status at the index date as well as any subsequent changes in exposure status (analogous to a per protocol analysis)

For all sections of this analysis individuals will be analysed according to a per protocol analysis. We will therefore replicate each of the steps outlined for objective 1i but curtail follow up time for individuals at the point at which their statin exposure status changes. We will censor individuals who were unexposed during the initial six month exposure period, but later received a statin prescription, at the date at which they were prescribed a statin. For individuals who were prescribed a statin during the initial six month exposure period and had a gap of >90 days in statin prescriptions, we will censor individuals at the point at which the prescription runs out. This will be estimated by calculating the last statin prescription date plus the number of days of medication prescribed (which will be estimated using data on pack size and dosage value).

Objective 2) To calculate effect estimates for the association between statin prescribing and i) all-cause mortality, ii) combined fatal and non-fatal coronary heart disease events and iii) combined fatal and non-fatal stroke events

We will repeat the steps undertaken for objective 1i but using the following outcomes: i) all-cause mortality, ii) fatal and non-fatal CHD events (and report separate estimates for myocardial infarction), iii) fatal and non-fatal stroke, iv) change in cholesterol at 1 and 2 years after baseline.

Supplementary Analyses

We plan to undertake the some supplementary analyses, for which our study is unlikely to have adequate power to formally assess, but which it would be useful to explore. We wish to explore whether statins have a differential effect on prevention of CVD within strata of: statin type (particularly when restricted to statins commonly used for primary prevention), SMI diagnosis (schizophrenia versus bipolar disorder), gender, and baseline CVD risk (e.g. >10% estimated 10 year CVD risk).

Bias and limitations

THIN offers a large, versatile and economical resource for examining real life patterns of primary care over time, and this data source has already been used by a number of studies investigating SMI and CVD.[51;55-57] However, THIN data are obtained for purposes of clinical management and estimates of effect may be inaccurate if confounding by indication and missing data are not correctly handled. Although the design developed by Danaei should help to select an unexposed group that is more similar to the exposed group than other study designs, confounding by indication is likely to remain an issue because statins are prescribed on the basis of clinical need rather than random allocation. Although it is not possible to fully replicate randomisation (i.e. approximately equal distribution of measured and unmeasured confounders across study arms), differences in measured confounding across the two groups can adjusted for using standard regression methods. We are not able to account for differences in unmeasured confounding, which cannot currently be adequately addressed by any methodology. However, we believe that our methods will perform at least as well as other possible study designs that have used primary care data to investigate the causal effects of statins.[36;39;40]

Reference List

(1) Osborn DP, Levy G, Nazareth I, Petersen I, Islam A, King MB. Relative risk of cardiovascular and cancer mortality in people with severe mental illness from the United Kingdom's General Practice Research Database. *Arch Gen Psychiatry* 2007; 64(2):242-249.

(2) Hennekens CH, Hennekens AR, Hollar D, Casey DE. Schizophrenia and increased risks of cardiovascular disease. *Am Heart J* 2005; 150(6):1115-1121.

(3) Tiihonen J, Lonnqvist J, Wahlbeck K, Klaukka T, Niskanen L, Tanskanen A et al. 11-year follow-up of mortality in patients with schizophrenia: a population-based cohort study (FIN11 study). *Lancet* 2009; 374(9690):620-627.

(4) Osborn DP, Wright CA, Levy G, King MB, Deo R, Nazareth I. Relative risk of diabetes, dyslipidaemia, hypertension and the metabolic syndrome in people with severe mental illnesses: systematic review and metaanalysis. *BMC Psychiatry* 2008; 8:84.

(5) Newcomer JW. Second-generation (atypical) antipsychotics and metabolic effects: a comprehensive literature review. *CNS Drugs* 2005; 19 Suppl 1:1-93.

(6) Cohen D. Atypical antipsychotics and new onset diabetes mellitus. An overview of the literature. *Pharmacopsychiatry* 2004; 37(1):1-11.

(7) Lamberti JS, Olson D, Crilly JF, Olivares T, Williams GC, Tu X et al. Prevalence of the metabolic syndrome among patients receiving clozapine. *Am J Psychiatry* 2006; 163(7):1273-1276.

(8) Buhagiar K, Parsonage L, Osborn DP. Physical health behaviours and health locus of control in people with schizophrenia-spectrum disorder and bipolar disorder: a cross-sectional comparative study with people with non-psychotic mental illness. *BMC Psychiatry* 2011; 11:104.

(9) Mitchell AJ, Lord O. Do deficits in cardiac care influence high mortality rates in schizophrenia? A systematic review and pooled analysis. *J Psychopharmacol* 2010; 24(4 Suppl):69-80.

(10) Mitchell AJ, Lord O, Malone D. Differences in the prescribing of medication for physical disorders in individuals with v. without mental illness: meta-analysis. *Br J Psychiatry* 2012; 201(6):435-443.

(11) National Institute for Health and Clinical Excellence. Lipid modification: Cardiovascular risk assessment and the modification of blood lipids for the primary and secondary prevention of cardiovascular disease. National Clinical Practice Guideline Number 67. 2014. London, UK.

(12) Gutierrez J, Ramirez G, Rundek T, Sacco RL. Statin therapy in the prevention of recurrent cardiovascular events: a sex-based meta-analysis. *Arch Intern Med* 2012; 172(12):909-919.

(13) Baigent C, Keech A, Kearney PM, Blackwell L, Buck G, Pollicino C et al. Efficacy and safety of cholesterol-lowering treatment: prospective meta-analysis of data from 90,056 participants in 14 randomised trials of statins. *Lancet* 2005; 366(9493):1267-1278.

(14) MRC/BHF Heart Protection Study of cholesterol lowering with simvastatin in 20,536 high-risk individuals: a randomised placebo-controlled trial. *Lancet* 2002; 360(9326):7-22.

(15) Lazar LD, Pletcher MJ, Coxson PG, Bibbins-Domingo K, Goldman L. Cost-effectiveness of statin therapy for primary prevention in a low-cost statin era. *Circulation* 2011; 124(2):146-153.

(16) Taylor F, Ward K, Moore TH, Burke M, Davey SG, Casas JP et al. Statins for the primary prevention of cardiovascular disease. *Cochrane Database Syst Rev* 2011;(1):CD004816.

(17) National Institute for Health and Clinical Excellence. Lipid modification: Cardiovascular risk assessment and the modification of blood lipids for the primary and secondary prevention of cardiovascular disease. National Clinical Practice Guideline Number 67. 2008. London, UK.

(18) National Institute for Health Clinical Excellence. Schizophrenia: Core Interventions in the Treatment and Management of Schizophrenia in Primary and Secondary Care. National Clinical Practice Guideline Number 82. 2009. London, UK.

(19) National Institute for Health and Clinical Excellance. Bipolar Disorder: The Management of Bipolar Disorder in Adults Children and Adolescents in Primary Care. National Clinical Practice Guideline Number 38. 2006. London, UK.

(20) Osterberg L, Blaschke T. Adherence to medication. *N Engl J Med* 2005; 353(5):487-497.

(21) Maningat P, Gordon BR, Breslow JL. How do we improve patient compliance and adherence to long-term statin therapy? *Curr Atheroscler Rep* 2013; 15(1):291.

(22) Ruokoniemi P, Sund R, Arffman M, Helin-Salmivaara A, Huupponen R, Keskimaki I et al. Are statin trials in diabetes representative of real-world diabetes care: a population-based study on statin initiators in Finland. *BMJ Open* 2014; 4(6):e005402.

(23) Nasrallah HA. Atypical antipsychotic-induced metabolic side effects: insights from receptor-binding profiles. *Mol Psychiatry* 2008; 13(1):27-35.

(24) Raeder MB, Ferno J, Vik-Mo AO, Steen VM. SREBP activation by antipsychotic- and antidepressant-drugs in cultured human liver cells: relevance for metabolic side-effects? *Mol Cell Biochem* 2006; 289(1-2):167-173.

(25) Ferno J, Raeder MB, Vik-Mo AO, Skrede S, Glambek M, Tronstad KJ et al. Antipsychotic drugs activate SREBP-regulated expression of lipid biosynthetic genes in cultured human glioma cells: a novel mechanism of action? *Pharmacogenomics J* 2005; 5(5):298-304.

(26) Ridker PM. Rosuvastatin in the primary prevention of cardiovascular disease among patients with low levels of low-density lipoprotein cholesterol and elevated high-sensitivity C-reactive protein: rationale and design of the JUPITER trial. *Circulation* 2003; 108(19):2292-2297.

(27) Shepherd J, Cobbe SM, Ford I, Isles CG, Lorimer AR, MacFarlane PW et al. Prevention of coronary heart disease with pravastatin in men with hypercholesterolemia. West of Scotland Coronary Prevention Study Group. *N Engl J Med* 1995; 333(20):1301-1307.

(28) Beishuizen ED, Jukema JW, Tamsma JT, van de Ree MA, van der Vijver JC, Putter H et al. No effect of statin therapy on silent myocardial ischemia in patients with type 2 diabetes without manifest cardiovascular disease. *Diabetes Care* 2005; 28(7):1675-1679.

(29) Nakamura H, Arakawa K, Itakura H, Kitabatake A, Goto Y, Toyota T et al. Primary prevention of cardiovascular disease with pravastatin in Japan (MEGA Study): a prospective randomised controlled trial. *Lancet* 2006; 368(9542):1155-1163.

(30) Knopp RH, d'Emden M, Smilde JG, Pocock SJ. Efficacy and safety of atorvastatin in the prevention of cardiovascular end points in subjects with type 2 diabetes: the Atorvastatin Study for Prevention of Coronary Heart Disease Endpoints in non-insulin-dependent diabetes mellitus (ASPEN). *Diabetes Care* 2006; 29(7):1478-1485.

(31) Baldassarre D, Veglia F, Gobbi C, Gallus G, Ventura A, Crepaldi G et al. Intima-media thickness after pravastatin stabilizes also in patients with moderate to no reduction in LDL-cholesterol levels: the carotid atherosclerosis Italian ultrasound study. *Atherosclerosis* 2000; 151(2):575-583.

(32) De Hert M., Kalnicka D, van WR, Wampers M, Hanssens L, Van ED et al. Treatment with rosuvastatin for severe dyslipidemia in patients with schizophrenia and schizoaffective disorder. *J Clin Psychiatry* 2006; 67(12):1889-1896.

(33) Joint Formulary Committee. British National Formulary. 2012. London, BMJ Group and Pharmaceutical Press.

(34) Seeger JD, Kurth T, Walker AM. Use of propensity score technique to account for exposure-related covariates: an example and lesson. *Med Care* 2007; 45(10 Supl 2):S143-S148.

(35) Danaei G, Tavakkoli M, Hernan MA. Bias in observational studies of prevalent users: lessons for comparative effectiveness research from a meta-analysis of statins. *Am J Epidemiol* 2012; 175(4):250-262.

(36) Danaei G, Rodriguez LA, Cantero OF, Logan R, Hernan MA. Observational data for comparative effectiveness research: an emulation of randomised trials of statins and primary prevention of coronary heart disease. *Stat Methods Med Res* 2013; 22(1):70-96.

(37) Hernan MA, Alonso A, Logan R, Grodstein F, Michels KB, Willett WC et al. Observational studies analyzed like randomized experiments: an application to postmenopausal hormone therapy and coronary heart disease. *Epidemiology* 2008; 19(6):766-779.

(38) Robins JM, Hernan MA. Estimation of the causal effects of time-varying exposures. In: Fitzmaurice G, Davidian M, Verbeke G, Molenberghs G, editors. Chapman & Hall; 2008.

(39) Smeeth L, Douglas I, Hall AJ, Hubbard R, Evans S. Effect of statins on a wide range of health outcomes: a cohort study validated by comparison with randomized trials. *Br J Clin Pharmacol* 2009; 67(1):99-109.

(40) Hippisley-Cox J, Coupland C. Unintended effects of statins in men and women in England and Wales: population based cohort study using the QResearch database. *BMJ* 2010; 340:c2197.

(41) Sterne JA, White IR, Carlin JB, Spratt M, Royston P, Kenward MG et al. Multiple imputation for missing data in epidemiological and clinical research: potential and pitfalls. *BMJ* 2009; 338:b2393.

(42) Nevalainen J, Kenward MG, Virtanen SM. Missing values in longitudinal dietary data: a multiple imputation approach based on a fully conditional specification. *Stat Med* 2009; 28(29):3657-3669.

(43) Welch C, Bartlett J. Application of multiple imputation using the two-fold fully conditional specification algorithm in longitudinal clinical data. *The Stata Journal* 2013.

(44) D'Agostino RB, Rubin DB. Estimating and using propensity scores with partially missing data. *Journal of the American Statistical Association* 2000; 95(451):749-759.

(45) Hayes JR, Groner JI. Using multiple imputation and propensity scores to test the effect of car seats and seat belt usage on injury severity from trauma registry data. *J Pediatr Surg* 2008; 43(5):924-927.

(46) Rosenbaum PR, Rubin DB. The Central Role of the Propensity Score in Observational Studies for Causal Effects. *Biometrika* 1983; 70(1):41-55.

(47) Shah BR, Laupacis A, Hux JE, Austin PC. Propensity score methods gave similar results to traditional regression modeling in observational studies: a systematic review. *Journal of Clinical Epidemiology* 2005; 58(6):550-559.

(48) Langley TE, Szatkowski L, Gibson J, Huang Y, McNeill A, Coleman T et al. Validation of The Health Improvement Network (THIN) primary care database for monitoring prescriptions for smoking cessation medications. *Pharmacoepidemiol Drug Saf* 2010; 19(6):586-590.

(49) Lewis JD, Schinnar R, Bilker WB, Wang X, Strom BL. Validation studies of the health improvement network (THIN) database for pharmacoepidemiology research. *Pharmacoepidemiol Drug Saf* 2007; 16(4):393-401.

(50) Horsfall L, Walters K, Petersen I. Identifying periods of acceptable computer usage in primary care research databases. *Pharmacoepidemiol Drug Saf* 2012.

(51) Osborn DP, Baio G, Walters K, Petersen I, Limburg H, Raine R et al. Inequalities in the provision of cardiovascular screening to people with severe mental illnesses in primary care: cohort study in the United Kingdom THIN Primary Care Database 2000-2007. *Schizophr Res* 2011; 129(2-3):104-110.

(52) White IR, Royston P. Imputing missing covariate values for the Cox model. *Statistics in Medicine* 2009; 28(15):1982-1998.

(53) D'Agostino RB, Sr., Vasan RS, Pencina MJ, Wolf PA, Cobain M, Massaro JM et al. General cardiovascular risk profile for use in primary care: the Framingham Heart Study. *Circulation* 2008; 117(6):743-753.

(54) DerSimonian R, Laird N. Meta-analysis in clinical trials. *Control Clin Trials* 1986; 7(3):177-188.

(55) Prah P, Petersen I, Nazareth I, Walters K, Osborn D. National changes in oral antipsychotic treatment for people with schizophrenia in primary care between 1998 and 2007 in the United Kingdom. *Pharmacoepidemiol Drug Saf* 2012; 21(2):161-169.

(56) Hayes J, Prah P, Nazareth I, King M, Walters K, Petersen I et al. Prescribing trends in bipolar disorder: cohort study in the United Kingdom THIN primary care database 1995-2009. *PLoS One* 2011; 6(12):e28725.

(57) Hardoon S, Hayes J, Blackburn R, Petersen I, Walters K, Nazareth I et al. Recording of Severe Mental Illness in United Kingdom Primary Care, 2000-2010. *PLoS One* 2013.
