# Supplementary material for: Statin prescribing for people with severe mental illnesses: a staggered cohort study of ‘real-world’ impacts
Source: BMJ Open. 2017 Mar 7;7(3):e013154. doi: 10.1136/bmjopen-2016-013154 (PMC5353294; doi:10.1136/bmjopen-2016-013154)
Supplement: supplementary data [file bmjopen-2016-013154supp3.pdf]

<u>Multiple imputation of unobserved covariate data</u>

Selecting the duration of the exposure window used in the final analysis

Our original protocol specified using an exposure window of 6 months (i.e. initiating each cohort at 6 monthly intervals, thus creating 22 staggered cohorts). However, when an exposure window of 6 or 12 months was specified the imputation model would not converge for some of the time periods, reflecting issues of perfect prediction arising from the small number of CVD events within sub-groups of statin use and gender[1]. We therefore used an exposure window of 24 months in order to support imputing data for each of the cohorts.

Specifying and testing the imputation model used in the final analysis

Complete cases: Individuals were defined as complete cases if they had a record of systolic blood pressure, weight and total cholesterol concentration measured in the 12 months before the index date in addition to a record of height (at age 21 years or older) and smoking status recorded at any time point.

Imputation of missing data: Unobserved covariate data were estimated using multiple imputation (White et al., 2011) to generate ten imputed datasets for the full study population. Data were combined using Rubin's rules to calculate effect estimates and confidence intervals. The *MI suite* of commands in Stata was used to estimate missing covariate data and analyse the imputed datasets. Data were separately imputed by gender in each of the five cohorts because CVD risk differs markedly between men and women, even after accounting for other factors such as age [2]. The imputation model used mean time-varying data for each year from three years either side of the index date to estimate unobserved records for the baseline period (twelve months before the index date) Figure 1.

Figure 1: Temporal relationships between the index date, baseline year (BL), and imputation variables (up to three years before (Y-3) and after (Y+3) the index date).

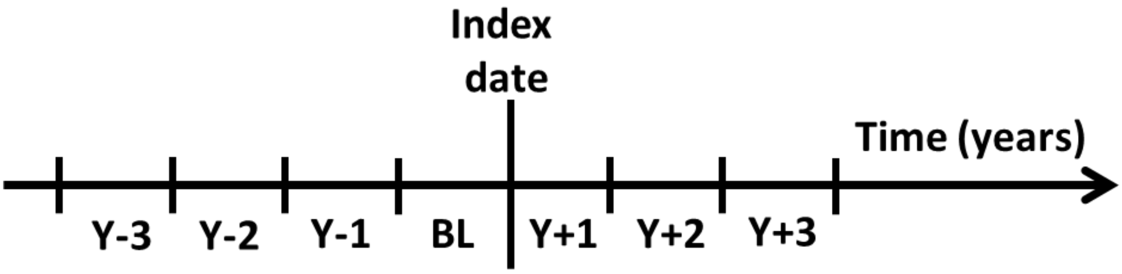

Final imputation model: *mi impute chained* (Stata version 14) was used to produce 10 imputed datasets for each cohort. The imputation model included measurements of total cholesterol, blood pressure, height and weight in log form.

Variables being imputed: log of cholesterol concentration at baseline and at +/-3 years, log of height, log of weight at baseline and +/-3 years, log of systolic blood pressure at baseline and +/- 3 years, Townsend quintile, smoking status. Although only baseline measurements were included in the analysis model a chained equations approach was used to impute data for the three years either side of baseline immediately prior to estimating missing baseline values.

Fully observed variables: statin exposure at the index date, CVD event indicator, Nelson-Aalen cumulative hazard estimates, indicators for statin prescribing at 1 and 2 years after the index date, sex, SMI diagnosis and baseline estimates of: 5 year age-band, diabetes status, heavy drinking, antihypertensive use, non-statin lipid modifying drug use, anti-depressant use, antipsychotic use and type/generation of antipsychotic, mood stabilizing drug use, quartiles of annual consultation rate, cancer diagnosis, hypothyroidism, familial hypercholesterolaemia, CKD, COPD, health authority region.

Developing the imputation model

Figure 2 describes the process used to specify the imputation model. The imputation model was developed using a forwards elimination approach starting with a basic model incorporating essential variables. Additional variables were added to this model in order of anticipated importance for patterns and values of missing data. Where the addition of a variable resulted in failure of the imputation model to converge it was omitted. The proportion of missing data for variables included in the Framingham risk score decreased over time: it was therefore anticipated that the earliest time periods would limit the complexity of the imputation model, relative to more recent data. For example, HDL-C was recorded for less

than 5% of individuals in 2002, but was available for over 60% of individuals in 2012. Similarly the pattern of recording for smoking status had changed over time such than in earlier years only current smokers had a record, whereas never- and ex-smoking statuses were much more commonly recorded for recent time points. The basic imputation model was therefore developed using data from the earliest cohorts and then applied to data from later cohorts.

Essential variables in the imputation model: As recommended[3], the imputation model included the outcome and all variables in the substantive model. The process outlined in Figure 2 started with a basic imputation model for each cohort, which included the following essential variables: age (in 5 year bands), statin prescribing at the index date and 1 and 2 years after the index date, diabetes status, systolic blood pressure, total cholesterol concentration, height, weight, smoking status, CVD events and the associated Nelson-Aalen cumulative hazard function estimate [4]. Time varying indicators marking whether an individual was prescribed a statin at one and two years after the index date were added to the imputation model. This improved the potential of the model to impute cholesterol concentration correctly at time periods after baseline in response to the initiation or cessation of statin therapy after the index date.

Additional variables in the imputation model: Other variables (termed $\boldsymbol{X_i}$ in Figure 2) that were identified from the literature as being correlated with CVD events and statin therapy were sequentially added to the model in order of anticipated importance. The order of importance was specified as follows: HDL-C, diastolic blood pressure, Townsend score, antihypertensive use, heavy drinking, non-statin lipid modifying drug use, antipsychotic use, antidepressant use, mood stabilising drug use, hypothyroidism, familial hypercholesterolaemia, consultation rate during baseline, cancer diagnoses, antipsychotic type and generation, CKD, COPD, asthma, atrial fibrillation, health authority region.

Figure 2: Flowchart for the process of developing the imputation model

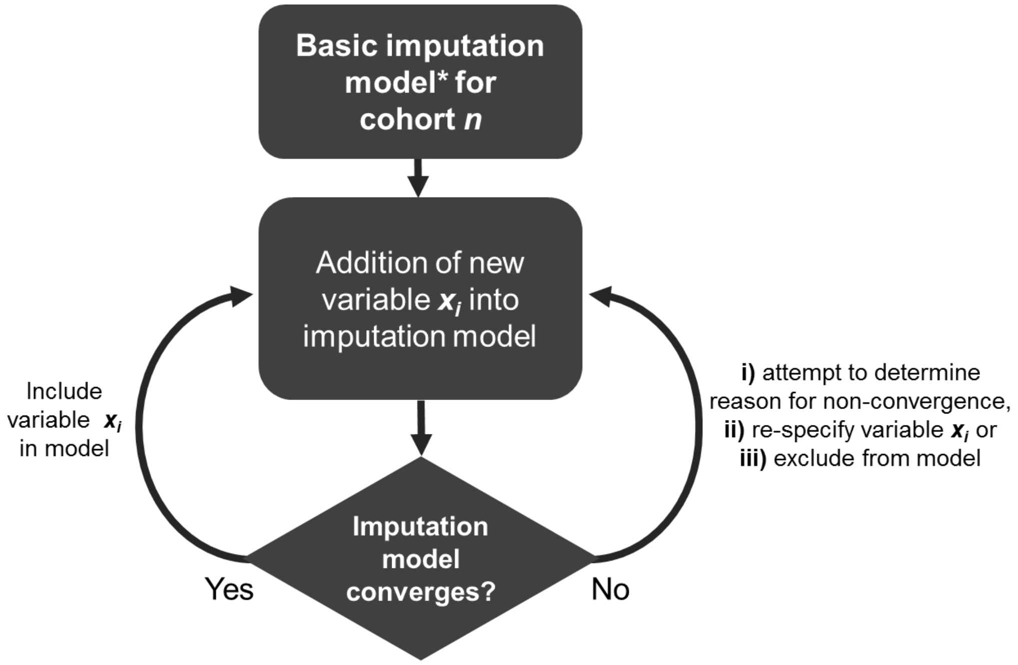

Review imputed values and – if the imputed values are plausible given the observed data - repeat the process for cohort *n+1* once all variables (x_i) have been added to the model

Cohort n denotes the cohort which was initiated at the earliest calendar time period (i.e. January 2002-December 2003).

Cohort n+1 denotes the next cohort in the sequence (e.g. January 2004-December 2005).

The **basic imputation model*** incorporated essential variables as follows: age (in 5 year bands), statin prescribing at the index date and 1 and 2 years after the index date, diabetes status, blood pressure, total cholesterol concentration, height, weight, smoking status, CVD events and the associated Nelson-Aalen cumulative hazard function estimate.

Each **additional variable (x_i)** was added in the following order: HDL-C, diastolic blood pressure and Townsend score for deprivation, antihypertensive use, heavy drinking, non-statin lipid modifying drug use, antipsychotic use, antidepressant use, mood stabilising drug use, hypothyroidism, familial hypercholesterolaemia, quartiles of consultation rate during baseline, cancer diagnoses, antipsychotic type and generation, CKD, COPD, asthma, atrial fibrillation, health authority region.

Imputation model checking: The standard output obtained from execution of *mi impute* in Stata was checked for any indication of model instability or misspecification. The plausibility of imputed values for total cholesterol, height, weight, systolic blood pressure and smoking status were carefully assessed for each cohort and across the full dataset (all five cohorts) as follows:

- Data that were imputed in log form were back-transformed in order to make assessment of the biological plausibility of these values easier to determine

- The range and distribution of imputed values was assessed relative to the observed (complete) data; particular attention was given to values of total cholesterol at baseline and other time points

- Complete and imputed data were also investigated by estimating CVD risk scores within strata of age (40-59, 60-69, 70-74, 75-84 years) and gender. In addition, the coefficients obtained for regression of estimated risk score on (continuous) age in years (separately for men and women) was used as a further means of gauging the compatibility of the complete and imputed datasets

- Multivariable regression models were used to assess the similarity of correlations within complete and imputed datasets between each of the variables included in the main analysis and statin prescribing or CVD events for the association. These associations were also useful for sense-checking complete and imputed data for well-established correlations such as increasing CVD risk and advancing age

Bibliography

[1] I. R. White, P. Royston, and A. M. Wood, "Multiple imputation using chained equations: Issues and guidance for practice," *Stat. Med.*, vol. 30, no. 4, pp. 377-399, Feb.2011.

[2] D. P. Osborn, S. Hardoon, R. Z. Omar, R. I. Holt, M. King, J. Larsen, L. Marston, R. W. Morris, I. Nazareth, K. Walters, and I. Petersen, "Cardiovascular risk prediction models for people with severe mental illness: results from the prediction and management of cardiovascular risk in people with severe mental illnesses (PRIMROSE) research program," *JAMA Psychiatry*, vol. 72, no. 2, pp. 143-151, Feb.2015.

[3] J. A. Sterne, I. R. White, J. B. Carlin, M. Spratt, P. Royston, M. G. Kenward, A. M. Wood, and J. R. Carpenter, "Multiple imputation for missing data in epidemiological and clinical research: potential and pitfalls," *BMJ*, vol. 338, p. b2393, 2009.

[4] I. R. White and P. Royston, "Imputing missing covariate values for the Cox model," *Statistics in Medicine*, vol. 28, no. 15, pp. 1982-1998, July2009.
