# Supplementary material for: Statin prescribing for people with severe mental illnesses: a staggered cohort study of ‘real-world’ impacts
Source: BMJ Open. 2017 Mar 7;7(3):e013154. doi: 10.1136/bmjopen-2016-013154 (PMC5353294; doi:10.1136/bmjopen-2016-013154)
Supplement: supplementary data [file bmjopen-2016-013154supp4.pdf]

Regression model outputs

Table 1: Output from multivariable logistic regression model for associations between covariates and either statin prescribing or CVD events in complete cases

COMPLETE CASES		Statin Prescribing			CVD events		
		OR	95% CI		OR	95% CI	
Age band	40-44	1.00	--	--	1.00	--	--
	45-49	0.97	0.75	1.24	3.64	1.66	7.95
	50-54	1.54	1.22	1.96	3.81	1.76	8.28
	55-59	1.98	1.56	2.53	4.82	2.24	10.36
	60-64	1.93	1.50	2.48	5.87	2.73	12.64
	65-69	2.19	1.66	2.87	8.43	3.92	18.13
	70-74	1.85	1.35	2.52	10.80	4.94	23.64
	75-79	2.04	1.42	2.93	9.74	4.21	22.57
	80-84	0.96	0.59	1.57	11.45	4.61	28.40
Sex	Male	1.00	--	--	1.00	--	--
	Female	0.55	0.48	0.63	0.73	0.57	0.93
	Diabetes	5.11	4.42	5.90	1.29	1.00	1.66
	Cholesterol	2.59	2.43	2.77	1.08	0.98	1.20
	Systolic BP	1.01	1.01	1.02	1.02	1.01	1.03
	BMI	1.03	1.02	1.04	1.01	0.99	1.03
Smoking status	Never	1.00	--	--	1.00	--	--
	Ex	1.20	0.97	1.49	1.36	0.96	1.94
	Current	1.75	1.50	2.04	1.33	1.00	1.77
SMI type	Bipolar	1.00	--	--	1.00	--	--
	Schizophrenia	1.00	0.86	1.16	0.96	0.73	1.26
Cohort	2002/3	1.00	--	--	1.00	--	--
	2004/5	2.18	1.64	2.89	0.68	0.47	0.98
	2006/7	2.29	1.74	3.00	0.52	0.36	0.75
	2008/9	1.75	1.33	2.30	0.33	0.22	0.50
	2009/10	1.16	0.89	1.53	0.16	0.10	0.25
Consultation rate	<4	1.00	--	--	1.00	--	--
	4-6	1.14	0.89	1.47	1.23	0.73	2.08
	7-12	1.26	0.99	1.60	1.26	0.77	2.07
	>12	1.38	1.08	1.76	1.40	0.85	2.30
Antipsychotic	None	1.00	--	--	1.00	--	--
	1	0.95	0.80	1.13	0.95	0.70	1.28
	2	0.97	0.83	1.12	0.97	0.73	1.28
Townsend quintile	1	1.00	--	--	1.00	--	--
	2	0.99	0.79	1.24	1.09	0.73	1.63
	3	1.01	0.82	1.26	1.56	1.07	2.27
	4	0.93	0.75	1.15	1.05	0.71	1.55
	5	1.16	0.93	1.44	1.00	0.66	1.50
	Antihypertensive	1.52	1.31	1.75	0.86	0.66	1.10
	Antidepressant	1.23	1.07	1.40	1.63	1.29	2.08
	Mood stabiliser	0.86	0.73	1.00	1.07	0.81	1.42

COMPLETE CASES		Statin Prescribing			CVD events		
		OR	95% CI		OR	95% CI	
	**Asthma**	1.09	0.89	1.34	1.37	0.96	1.94
	**COPD**	1.09	0.78	1.51	2.35	1.55	3.56
	**FH**	10.27	7.09	14.87	1.16	0.66	2.03
	**AF**	0.98	0.52	1.84	0.58	0.17	1.94
	**Cancer**	0.61	0.36	1.04	0.65	0.26	1.65
	**Hypothyroidism**	0.99	0.55	1.80	0.48	0.11	2.05
	**CKD**	0.87	0.67	1.13	1.22	0.79	1.87
	**Heavy drinking**	0.77	0.64	0.92	0.59	0.40	0.86
	**Non statin use**	1.11	0.68	1.82	0.97	0.38	2.49

Table 2: Output from multivariable logistic regression model for associations between covariates and either statin prescribing or CVD events in the full dataset (with imputed values)

IMPUTED DATA		Statin Prescribing			CVD events		
		OR	95% CI		OR	95% CI	
	**40-44**	1.00	--	--	1.00	--	--
	**45-49**	1.18	0.98	1.42	2.00	1.57	2.56
	**50-54**	1.66	1.40	1.98	2.47	1.94	3.15
	**55-59**	2.11	1.77	2.51	3.21	2.53	4.07
**Age band**	**60-64**	2.41	2.01	2.88	4.43	3.49	5.61
	**65-69**	2.71	2.24	3.28	6.18	4.87	7.85
	**70-74**	2.30	1.85	2.85	7.64	5.97	9.77
	**75-79**	1.78	1.39	2.28	7.05	5.42	9.16
	**80-84**	0.98	0.70	1.37	7.82	5.89	10.38
**Sex**	**Male**	1.00	--	--	1.00	--	--
	**Female**	0.56	0.50	0.61	0.71	0.64	0.79
	**Diabetes**	7.20	6.49	7.99	1.31	1.13	1.51
	**Cholesterol**	2.25	2.15	2.36	1.06	1.00	1.13
	**Systolic BP**	1.01	1.01	1.02	1.01	1.01	1.01
	**BMI**	1.04	1.04	1.05	1.01	1.00	1.02
	**Never**	1.00	--	--	1.00	--	--
**Smoking status**	**Ex**	1.27	1.10	1.47	1.12	0.96	1.30
	**Current**	1.58	1.41	1.76	1.38	1.23	1.54
**SMI type**	**Bipolar**	1.00	--	--	1.00	--	--
	**Schizophrenia**	1.01	0.91	1.13	0.83	0.74	0.93
	**2002/3**	1.00	--	--	1.00	--	--
	**2004/5**	2.12	1.78	2.53	0.71	0.63	0.81
**Cohort**	**2006/7**	2.84	2.39	3.37	0.48	0.42	0.56
	**2008/9**	2.49	2.09	2.97	0.33	0.28	0.38
	**2009/10**	2.15	1.80	2.57	0.15	0.13	0.19
	**<4**	1.00	--	--	1.00	--	--
**Consultation rate**	**4-6**	1.79	1.53	2.11	1.01	0.87	1.17
	**7-12**	2.34	2.00	2.73	1.10	0.96	1.27

IMPUTED DATA		Statin Prescribing			CVD events		
		OR	95% CI		OR	95% CI	
	**>12**	2.87	2.46	3.35	1.10	0.95	1.27
**Antipsychotic**	**None**	1.00	--	--	1.00	--	--
	**1**	0.94	0.84	1.06	1.01	0.89	1.14
	**2**	1.11	1.00	1.23	1.05	0.94	1.18
**Townsend quintile**	**1**	1.00	--	--	1.00	--	--
	**2**	0.96	0.82	1.12	1.21	1.03	1.42
	**3**	1.01	0.87	1.18	1.41	1.21	1.65
	**4**	0.91	0.78	1.06	1.22	1.04	1.43
	**5**	1.13	0.97	1.32	1.27	1.07	1.50
	**Antihypertensive**	2.35	2.12	2.60	1.31	1.17	1.47
	**Antidepressant**	0.96	0.83	1.11	1.18	1.00	1.38
	**Mood stabiliser**	1.04	0.83	1.31	1.15	0.93	1.43
	**Asthma**	1.03	0.94	1.13	1.12	1.01	1.23
	**COPD**	0.85	0.76	0.94	1.00	0.89	1.12
	**FH**	38.39	28.99	50.83	1.42	0.95	2.12
	**AF**	1.22	0.83	1.79	1.04	0.71	1.53
	**Cancer**	0.64	0.43	0.94	0.69	0.46	1.02
	**Hypothyroidism**	1.14	0.74	1.76	1.21	0.76	1.94
	**CKD**	1.29	1.06	1.57	1.17	0.90	1.51
	**Heavy drinking**	0.95	0.83	1.07	0.98	0.85	1.14
	**Non statin use**	1.81	1.24	2.66	0.99	0.51	1.89

Table 3: Analysis of complete case data in the main analysis. Estimates presented alongside imputed data results

Section of Main Analysis		N=6915 Complete case analysis			N=45824 Imputed data analysis		
**VARIABLES IN THE ADJUSTED MODEL**		**IRR**	**95% CI**		**IRR**	**95% CI**	
**Impact of adding additional variables into the model**	**Crude**	1.11	0.78	1.58	1.39	1.11	1.74
	**Age + Sex**	1.06	0.75	1.51	1.16	0.93	1.46
	**Diabetes**	1.01	0.70	1.44	1.04	0.82	1.31
	**Cholesterol**	1.06	0.72	1.56	0.98	0.77	1.25
	**BMI**	1.03	0.70	1.53	0.96	0.75	1.22
	**Sys BP**	0.99	0.67	1.46	0.92	0.72	1.18
	**Smoker**	0.96	0.65	1.42	0.89	0.70	1.14
	**SMI type**	0.96	0.65	1.43	0.89	0.70	1.14
	**Year**	0.92	0.62	1.37	0.92	0.72	1.18
	**Fully adjusted***	**0.88**	**0.58**	**1.36**	**0.89**	**0.68**	**1.15**
**COHORT-SPECIFIC ESTIMATES**		**IRR**	**95% CI**		**IRR**	**95% CI**	
**Cohort (fully adjusted*)**	**2002/3**	0.41	0.02	7.08	0.68	0.35	1.32
	**2004/5**	0.91	0.40	2.06	1.19	0.75	1.88
	**2006/7**	0.98	0.43	2.24	0.99	0.58	1.69
	**2008/9**	0.71	0.24	2.07	0.70	0.35	1.41
	**2010/11**	2.70	0.64	8.35	0.77	0.26	2.31
**PRIMARY OUTCOME**		**IRR**	**95% CI**		**IRR**	**95% CI**	
**(fully adjusted*)**	**MI and Stroke**	0.88	0.58	1.36	0.89	0.68	1.15
**SECONDARY OUTCOMES**		**IRR**	**95% CI**		**IRR**	**95% CI**	
**(fully adjusted*)**	**All-cause mortality**	1.02	0.82	1.27	0.89	0.78	1.02
	**Stroke**	0.85	0.49	1.48	0.96	0.68	1.34
	**MI**	0.93	0.47	1.83	0.75	0.48	1.15
**SUB-GROUP ANALYSES (fully adjusted*)**		**IRR**	**95% CI**		**IRR**	**95% CI**	
**SMI type**	**Bipolar disorder**	0.78	0.38	1.61	0.89	0.60	1.32
	**Schizophrenia**	0.90	0.53	1.52	0.85	0.60	1.20
**Gender**	**Men**	0.95	0.53	1.72	0.96	0.65	1.42
	**Women**	0.86	0.46	1.61	0.84	0.58	1.20
**CVD risk strata**	**≥10%**	0.96	0.60	1.53	0.93	0.69	1.26
	**≥15%**	1.03	0.65	1.62	0.90	0.67	1.21
	**≥20%**	1.00	0.63	1.60	0.82	0.59	1.13
	**≥25%**	0.91	0.55	1.50	0.73	0.52	1.05

* Final model covariates (baseline): age and sex, diabetes, total cholesterol concentration, BMI, systolic blood pressure (Sys BP), smoking status, SMI type, cohort time period, antihypertensive use, antidepressant use, quartile of consultation rate, antipsychotic use and type, mood-stabiliser use, Townsend score, asthma, COPD, hypothyroidism, CKD, AF, familial hypercholesterolaemia, heavy drinking, non-statin lipid modification, cancer

Table 4: Output from multivariable linear regression model for associations between statin prescribing and total cholesterol at 1 year after the index date (complete cases)

Factor		Coefficient	Std. Err.	P>t	95% CI	
**Statin prescription**		-1.29	0.03	<0.001	-1.35	-1.23
**Cholesterol at baseline**		-0.36	0.01	<0.001	-0.39	-0.34
**Age band**	**40-44**	baseline	--	--	--	
	**45-49**	-0.05	0.04	0.26	-0.13	0.04
	**50-54**	-0.05	0.04	0.25	-0.13	0.03
	**55-59**	-0.02	0.05	0.70	-0.11	0.07
	**60-64**	-0.06	0.05	0.17	-0.15	0.03
	**65-69**	-0.13	0.05	0.01	-0.23	-0.03
	**70-74**	-0.11	0.06	0.05	-0.22	0.00
	**75-79**	-0.20	0.07	<0.001	-0.33	-0.07
	**80-84**	-0.13	0.08	0.10	-0.27	0.02
**Sex**	**Male**	baseline	--	--	--	
	**Female**	0.09	0.02	<0.001	0.05	0.14
	**Diabetes**	-0.11	0.03	<0.001	-0.16	-0.06
	**Systolic BP**	0.00	0.00	0.17	0.00	0.00
	**BMI**	0.00	0.00	0.05	-0.01	0.00
**Smoking status**	**Never**	baseline	--	--	--	
	**Ex**	-0.02	0.04	0.53	-0.10	0.05
	**Current**	0.02	0.03	0.51	-0.04	0.07
**SMI type**	**Bipolar**	baseline	--	--	--	
	**Schizophrenia**	-0.04	0.03	0.19	-0.09	0.02
**Cohort**	**2002/3**	baseline	--	--	--	
	**2004/5**	-0.11	0.06	0.07	-0.22	0.01
	**2006/7**	-0.11	0.06	0.05	-0.23	0.00
	**2008/9**	-0.06	0.06	0.33	-0.17	0.06
	**2009/10**	-0.05	0.06	0.34	-0.16	0.06
**Consultation rate**	**<4**	baseline	--	--	--	
	**4-6**	0.00	0.05	0.95	-0.09	0.10
	**7-12**	-0.03	0.04	0.53	-0.12	0.06
	**>12**	-0.01	0.04	0.80	-0.10	0.08
**Antipsychotic**	**None**	baseline	--	--	--	
	**1**	-0.05	0.03	0.16	-0.11	0.02
	**2**	-0.06	0.03	0.03	-0.12	-0.01
**Townsend quintile**	**1**	baseline	--	--	--	
	**2**	-0.02	0.04	0.67	-0.10	0.06
	**3**	-0.07	0.04	0.07	-0.15	0.00
	**4**	-0.08	0.04	0.04	-0.15	0.00
	**5**	-0.03	0.04	0.42	-0.11	0.04
	**Antihypertensive**	-0.05	0.03	0.06	-0.10	0.00
	**Antidepressant**	0.03	0.02	0.20	-0.02	0.08
	**Mood stabiliser**	0.03	0.03	0.33	-0.03	0.08

Factor		Coefficient	Std. Err.	P>t	95% CI	
	**Asthma**	0.08	0.04	0.03	0.01	0.15
	**COPD**	-0.03	0.05	0.53	-0.14	0.07
	**FH**	-0.06	0.07	0.42	-0.21	0.09
	**AF**	0.00	0.12	1.00	-0.24	0.24
	**Cancer**	-0.05	0.11	0.67	-0.26	0.17
	**Hypothyroidism**	-0.24	0.10	0.02	-0.44	-0.04
	**CKD**	-0.02	0.04	0.64	-0.10	0.06
	**Heavy drinking**	0.04	0.03	0.22	-0.02	0.10
	**Non statin use**	0.20	0.10	0.05	0.00	0.39
	**Model intercept**	1.96	0.16	<0.001	1.64	2.27

Table 5: Output from multivariable linear regression model for associations between statin prescribing and total cholesterol at 2 years after the index date (complete cases)

Factor		Coefficient	Std. Err.	P>t	95% CI	
**Statin prescription**		-1.19	0.04	<0.001	-1.26	-1.12
**Cholesterol at baseline**		-0.41	0.01	<0.001	-0.44	-0.38
**Age band**	**40-44**	baseline	--	--	--	
	**45-49**	-0.01	0.05	0.86	-0.10	0.08
	**50-54**	-0.04	0.05	0.36	-0.13	0.05
	**55-59**	-0.01	0.05	0.79	-0.10	0.08
	**60-64**	-0.02	0.05	0.73	-0.11	0.08
	**65-69**	-0.15	0.05	0.01	-0.26	-0.04
	**70-74**	-0.19	0.06	<0.001	-0.31	-0.08
	**75-79**	-0.19	0.08	0.02	-0.35	-0.03
	**80-84**	-0.28	0.08	<0.001	-0.44	-0.12
**Sex**	**Male**	baseline	--	--	--	
	**Female**	0.14	0.03	<0.001	0.09	0.19
	**Diabetes**	-0.14	0.03	<0.001	-0.20	-0.08
	**Systolic BP**	0.00	0.00	0.03	0.00	0.00
	**BMI**	-0.01	0.00	<0.001	-0.01	0.00
**Smoking status**	**Never**	baseline	--	--	--	
	**Ex**	-0.05	0.04	0.18	-0.13	0.02
	**Current**	-0.06	0.03	0.05	-0.12	0.00
**SMI type**	**Bipolar**	baseline	--	--	--	
	**Schizophrenia**	-0.03	0.03	0.27	-0.09	0.03
**Cohort**	**2002/3**	baseline	--	--	--	
	**2004/5**	-0.08	0.07	0.26	-0.23	0.06
	**2006/7**	0.03	0.07	0.69	-0.11	0.17
	**2008/9**	0.04	0.07	0.57	-0.10	0.18
	**2009/10**	0.13	0.07	0.07	-0.01	0.26
**Consultation rate**	**<4**	baseline	--	--	--	
	**4-6**	0.03	0.05	0.55	-0.06	0.12

Factor		Coefficient	Std. Err.	P>t	95% CI	
	**7-12**	0.05	0.04	0.28	-0.04	0.13
	**>12**	0.04	0.04	0.31	-0.04	0.13
**Antipsychotic**	**None**	baseline	--	--	--	
	**1**	-0.06	0.04	0.07	-0.13	0.01
	**2**	-0.07	0.03	0.02	-0.13	-0.01
**Townsend quintile**	**1**	baseline	--	--	--	
	**2**	-0.01	0.04	0.78	-0.10	0.07
	**3**	-0.12	0.04	0.01	-0.20	-0.04
	**4**	-0.06	0.04	0.12	-0.14	0.02
	**5**	-0.07	0.04	0.09	-0.15	0.01
	**Antihypertensive**	-0.03	0.03	0.35	-0.09	0.03
	**Antidepressant**	0.02	0.03	0.51	-0.03	0.07
	**Mood stabiliser**	-0.03	0.03	0.35	-0.09	0.03
	**Asthma**	0.10	0.04	0.02	0.02	0.19
	**COPD**	-0.09	0.06	0.13	-0.22	0.03
	**FH**	0.04	0.09	0.63	-0.13	0.21
	**AF**	-0.08	0.10	0.43	-0.27	0.11
	**Cancer**	0.00	0.13	0.99	-0.25	0.25
	**Hypothyroidism**	-0.03	0.14	0.84	-0.31	0.25
	**CKD**	-0.03	0.04	0.54	-0.11	0.06
	**Heavy drinking**	0.04	0.04	0.30	-0.03	0.11
	**Non statin use**	0.08	0.09	0.37	-0.09	0.25
	**Model intercept**	2.05	0.17	<0.001	1.71	2.39
