# Supplementary material for: Statin prescribing for people with severe mental illnesses: a staggered cohort study of ‘real-world’ impacts
Source: BMJ Open. 2017 Mar 7;7(3):e013154. doi: 10.1136/bmjopen-2016-013154 (PMC5353294; doi:10.1136/bmjopen-2016-013154)
Supplement: supplementary data [file bmjopen-2016-013154supp5.pdf]

Estimating sample size for a staggered cohort study design

The minimum standard error of the incident rate ratio (IRR) is driven by Poisson counting error, which is equal to the square root of the mean (estimated as the number of events in statin users). The half width of the 95% CI around its central limit can therefore be estimated as 1.96 multiplied by the square root of the mean divided by the mean. For example, there were 83 cases of MI and stroke in statin users, the half width of the 95% CI can therefore be estimated as $1.96*(\sqrt{83}/83) = 0.22$ (or 22%). In this instance an IRR larger than 0.78 would be unlikely to be associated with a 95% confidence interval that excluded 1 (indicating a null effect).
